# A Pharmacist Consultant Service for Deprescribing Opioids and Benzodiazepines in Older Adults: A Cluster Randomized Trial

**DOI:** 10.1001/jamanetworkopen.2025.60581

**Published:** 2026-02-26

**Authors:** Jan Busby-Whitehead, Stefanie P. Ferreri, Joshua Niznik, Lori T. Armistead, Tamera Hughes, Liang Zhao, Benjamin Y. Urick, John S. Preisser, Claire Larson, Ellen Roberts, Leigh Foushee, Yara Haddad

**Affiliations:** 1 Division of Geriatric Medicine, Department of Medicine, University of North Carolina at Chapel Hill, School of Medicine, Chapel Hill; 2 UNC Center for Aging and Health, University of North Carolina at Chapel Hill, School of Medicine, Chapel Hill; 3 Division of Practice Advancement and Clinical Education, University of North Carolina at Chapel Hill, UNC Eshelman School of Pharmacy, Chapel Hill; 4 Division of Pharmaceutical Outcomes and Policy, University of North Carolina at Chapel Hill, Eshelman School of Pharmacy, Chapel Hill; 5 Now with Chiesi, USA, Cary, North Carolina.; 6 Now with Fred Wilson School of Pharmacy, High Point University, High Point, North Carolina.; 7 Department of Pharmacy Analytics, UNC Health, Chapel Hill, North Carolina; 8 Prime Therapeutics, Eagan, Minnesota; 9 Department of Biostatistics, Gillings School of Global Public Health, University of North Carolina at Chapel Hill, Chapel Hill; 10 Department of Pharmacy, UNC Health, Chapel Hill, North Carolina; 11 National Center for Injury Prevention and Control, Centers for Disease Control and Prevention, Atlanta, Georgia

## Abstract

**Importance:**

Opioid and benzodiazepine use by older adults is related to increased fall risk.

**Objective:**

To evaluate whether a centralized consultant pharmacist intervention increases opioid and benzodiazepine deprescribing and reduces falls among older adults in primary care.

**Design, Setting, and Participants:**

This intention-to-treat cluster randomized trial with 1-year follow-up included 2075 adults 65 years or older with long-term opioid use (n = 965) or benzodiazepine use (n = 1110) identified from electronic health record prescription data with a clinic visit from June 1, 2020, to July 31, 2022. Patients were from primary care clinics in the University of North Carolina Health Physician Network without embedded pharmacists, all using a shared electronic health record (EHR). Exclusions included active cancer treatment, dementia, or non–English-language preference. Statistical analysis was performed in November 2022.

**Intervention:**

Clinics were randomized to a centralized consultant pharmacist service or usual care. Pharmacists reviewed the EHR and the state’s prescription drug monitoring program and then provided patient-specific opioid or benzodiazepine tapering recommendations for prescribers at intervention clinics. Control clinics received no deprescribing support.

**Main Outcomes and Measures:**

Primary outcomes were average daily morphine milligram equivalents (MMEs) and diazepam milligram equivalents (DMEs) ordered in the year after the index visit. Secondary outcomes included medication discontinuation (≥180-day gap in orders) and incident falls identified using *International Classification of Diseases, Ninth Revision (ICD-9) *or* International Statistical Classification of Diseases *and* Related Health Problems, Tenth Revision (ICD-10) *codes.

**Results:**

A total of 2075 patients (mean [SD] age, 74.6 [0.13 for opioid group and 0.15 for benzodiazepine group] years; 1429 [68.9%] female) met criteria for long-term opioid or benzodiazepine use. Both intervention and control clinics showed reductions in opioid and benzodiazepine exposure, but between-group differences were not statistically significant. Mean (SD) daily MMEs decreased by 8.8% in intervention clinics (26.9 [54.2] to 24.5 [50.4]) and 5.4% in controls (18.7 [33.4] to 17.6 [32.0]) (*P* = .71); mean (SD) DMEs decreased by 11.4% (8.8 [10.4] to 7.8 [10.1]) and 1.5% (6.6 [8.2] to 6.5 [8.3]), respectively (*P* = .07). Although the proportion of patients who discontinued opioids was 21.4% in intervention clinics and 19.9% in control clinicals, this difference was not statistically significant (odds ratio [OR], 1.20; 95% CI, 0.85-1.71). The proportion of patients who discontinued benzodiazepines was 22.0% in intervention clinics and 18.4% in control clinics. This difference was not statistically significant (OR, 1.41; 95% CI, 0.94-2.03). There was no difference in the likelihood of incident falls between patients taking opioids in the control and intervention clinics (OR, 1.36; 95% CI, 0.94-1.96). In subgroup analyses, patients taking benzodiazepines with less than 10 daily DMEs had significantly reduced DMEs (effect size, −0.22; 95% CI, −0.36 to −0.08; *P* = .002).

**Conclusions and Relevance:**

This cluster randomized trial found that a centralized consultant pharmacist model was feasible, integrated well into primary care workflows, and resulted in high practitioner acceptance of recommendations; however, it did not significantly reduce opioid or benzodiazepine prescribing or falls within 1 year. These results suggest that more intensive or sustained deprescribing strategies may be needed to produce clinically meaningful reductions in high-risk medication use among older adults in primary care clinics.

**Trial Registration:**

ClinicalTrials.gov Identifier: NCT04272671

## Key Points

**Question:**

What impact does a pharmacist consultant service have on deprescribing rates of opioids and benzodiazepines in older adults?

**Findings:**

In this cluster randomized trial of 15 primary care clinics with 961 and 1107 older adults taking long-term opioids and benzodiazepines, respectively, reductions in morphine and diazepam milliequivalents were achieved in both the intervention and control groups. There was no statistically significant reduction in medication use or falls.

**Meaning:**

These results suggest that a consultant pharmacist–led intervention is feasible to implement in primary care clinics.

## Teaser

This cluster randomized trial of older adults taking long-term opioids and benzodiazepines examines whether an intervention that used a remote pharmacy team to create deprescribing recommendations reduced medication exposure and falls among this population.

## Introduction

Use of potentially inappropriate medications by older adults contributes to adverse drug events, falls, hospitalizations, and mortality.^1^ Deprescribing medications that are potentially inappropriate, particularly when used at high doses, for long durations, or in risky combinations, is a critical strategy to reduce preventable harm.^2,3,4,5^ Opioids and benzodiazepines are commonly prescribed and strongly associated with morbidity, mortality, and fall risk.^6,7,8^ Even when taken as prescribed, long-term use can impair balance and cognition and increase injurious falls, especially when both medications are used concurrently.^4,6,7,9,10,11^

Falls lead to approximately 3 million emergency department (ED) visits and 1 million hospitalizations annually.^12,13^ Medications that cause dizziness or somnolence further elevate this risk.^3,7,8,14,15^ Prior randomized studies have leveraged primary care or community pharmacists to screen for falls, using STEADI (Stopping Early Accidents, Deaths, and Injuries) tools, and address potentially inappropriate medication use.^16,17,18,19,20^ Those studies demonstrated high practitioner acceptance of most medication-related recommendations,^16,17,18,19,20^ but deprescribing opioids and benzodiazepines remains challenging in primary care.^21,22^ Most fall-related deprescribing intervention studies focus exclusively on benzodiazepines, with limited studies addressing opioids. Interventions have included educational brochures, medical record review, and pharmacist-led services. A recent review suggested that patient education combined with direct pharmacist engagement is most effective to change medication use.^23^ However, many studies are small, lack control groups, or are resource intensive.

Because many primary care clinics cannot afford embedded pharmacists, we developed a centralized consultant pharmacist model designed to provide cost-efficient deprescribing support.^24,25^ This model assists practitioners with tapering opioids and benzodiazepines while reducing workflow burden. Our clinic-randomized intervention used a remote pharmacy team to create patient-specific opioid and benzodiazepine deprescribing recommendations for older adults in primary care practices. The primary aim was to evaluate whether this intervention would reduce falls among older adults.

## Methods

### Overview

This cluster randomized trial in North Carolina evaluated whether consultant pharmacist recommendations could reduce opioid and benzodiazepine use in primary care settings.^26^ This study was approved by the University of North Carolina (UNC) institutional review board, which waived patient informed consent because clinics displayed institutional review board–required study flyers for all patients. Clinic medical directors provided written approval before randomization. This study followed the Consolidated Standards of Reporting Trials (CONSORT) reporting guideline. The trial protocol can be found in Supplement 1.

### Study Setting

**Figure 1.**
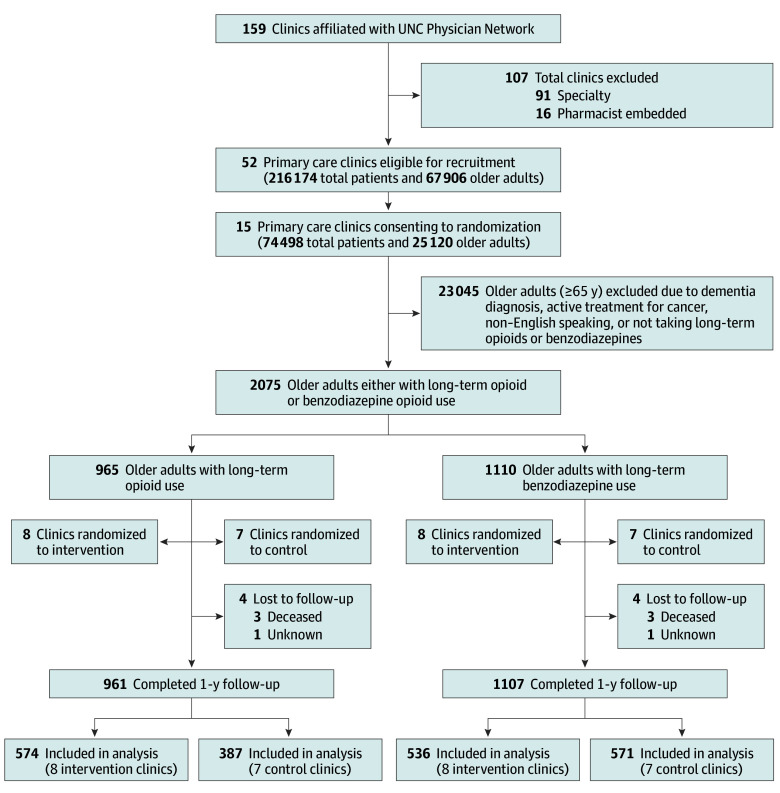
CONSORT Flowchart of Clinic and Patient Randomization UNC indicates University of North Carolina.

The study recruited primary care clinics within the UNC Health Physician Network, including more than 300 outpatient practitioners across 159 practices in 14 rural and urban or suburban counties. Of the 159 UNC Health Physician Network clinics, 107 were excluded because they had an embedded pharmacist or they were a specialty clinic. Fifty-two clinics were eligible for recruitment and 15 consented to participate. All clinics use Epic, the shared electronic health record (EHR) system. Recruitment occurred between December 1, 2019, and November 30, 2020. Recruitment ended when power calculations, based solely on baseline data and not observed follow-up treatment differences, demonstrated that the sample was sufficient for at least 80% power to detect a difference of at least 30% in average daily milligram equivalents prescribed. Clinic randomization and characteristics appear in Figure 1 and eTable 1 in Supplement 2.

In particular, power was based on 2-sided, 2-sample *t* tests at the 5% level, with the degrees of freedom based on the number of clusters. Specifically, with the use of nQuery software, version 7.0 (Statsols), a sample of 7 practices in the control and 8 in the intervention arm, with approximately 60 individuals per cluster and a coefficient of variation of cluster sizes of 0.7, achieved 80% and 90% power, respectively, to detect a difference of 30% in opioid (morphine milligram equivalents [MMEs]) and benzodiazepine (diazepam milligram equivalents [DMEs]) exposures. As outcomes on the untransformed scale were highly skewed, power was calculated on the natural log scale of MME and DME, respectively, to detect a −0.356 difference (or exp[−0.356] = 0.70 on the untransformed scale for a reduction of 30%) assuming that (for the planned analysis of covariance analysis) the baseline adjusted SD was 0.821 for MMEs and 0.757 for DMEs, whereas the intracluster correlations were 0.06 for MMEs and 0.05 DMEs. Parameter inputs were baseline mean log (unadjusted SD) MMEs of 2.50 (1.15) and DMEs of 1.54 (1.06) and adjusted squared SD (SD^2^[1 − *R*^2^]) with a multiple correlation coefficient *R* = 0.7.

### Sample and Data Sources

Primary care practitioners caring for patients 65 years and older with long-term use of prescription opioids and/or benzodiazepines and who had an upcoming primary care visit were included. The primary care visit served as the index date. Patients with long-term opioid and benzodiazepines use were identified based on prescription orders in the EHR from the prior year. Long-term use definitions were developed based on preliminary data analyses conducted by our research team described in our protocol.^26^ Long-term use was defined based on the best balance of sensitivity and specificity. This ultimately was defined as at least 4 opioid prescription orders in the prior year with at least 1 in the preceding 90 days for opioids (sensitivity. 88.9%; specificity, 64.8%) and for prescription orders in the prior year with at least 1 in the preceding 180 days for benzodiazepines (sensitivity, 81.8%; specificity, 48.7%). Patients with active cancer treatment or dementia based on *International Statistical Classification of Diseases and Related Health Problems, Tenth Revision (ICD-10) *codes,^27^ who were non–English speaking, or who had no evidence of any prescription orders (including opioids and benzodiazepines) in the year following their index appointment were excluded (4 patients taking opioids and 3 patients taking benzodiazepines).

### Intervention Description

Clinics were randomized (1:1) to intervention (n = 8) or control (n = 7) groups across 3 recruitment waves. Patients were enrolled for 8 to 12 months depending on clinic start date, with 1 year follow-up for all patients. Data collection ended on July 31, 2022.

The intervention used centralized consultant pharmacists through the Carolina Assessment of Medications Program team, which provides population health services for the UNC Health Care system. Carolina Assessment of Medications Program pharmacists reviewed eligible patients’ EHR data and the state’s prescription drug monitoring program and generated specific tapering recommendations for opioids and benzodiazepines. Recommendations included taper schedules, cross-taper plans, adjunctive medications, and alternative therapies. All recommendations were communicated in a progress note directly through the EHR. The recommendations were also sent as staff messages to the practitioners. The study website provided prescriber educational tools and patient educational materials, including brochures from the Centers for Disease Control and Prevention’s STEADI initiative. Control clinics received general patient education materials unrelated to deprescribing, opioids, or benzodiazepines.

Weekly automated reports identified eligible patients with upcoming appointments. Pharmacists reviewed the reports before each patient’s appointment. Pharmacists either provided a tapering recommendation or deemed patients ineligible for a recommendation. Reasons for ineligibility could be if treatment was for an appropriate indication or dosage reduction was inappropriate or unwarranted. Tapering recommendations for an opioid or benzodiazepine taper included detailed dosing schedules and potential adjunctive or replacement therapies. These were shared with the primary care practitioner to implement at the upcoming patient visit. EHR templates were created for initial recommendations, follow-up notes, patient-deemed ineligible notes, and documentation of patient or practitioner rejection of the recommendation.^28^ Notes for each type of recommendation had a unique title that was extractable as a discrete data field.

### Outcomes

The primary outcome was patient medication exposure during the 1-year postintervention period, calculated from EHR prescription orders. Average daily MMEs for opioids and DMEs for benzodiazepines were calculated for each patient using prescribed quantity, directions, and dose. Daily units were divided by the total quantity of pills ordered to determine the intended day’s supply. Daily units were then multiplied by the ordered dosage in milligrams from the medication name to obtain the daily milligram exposure. This total was then subsequently converted to average daily MMEs or DMEs. The assumption was that medication orders were filled on the date written, adjusting for overlapping supplies. The average daily exposure for each patient for 1 year was calculated by summing the average daily MMEs for each prescription order and dividing by 365 days.

As a secondary outcome, we examined the likelihood of opioid and benzodiazepine discontinuation in the 1-year period after the intervention, which was defined as a 180-day gap in medication orders. We also examined the impact of the intervention on incident falls throughout the 1-year follow-up period. Incident falls were identified based on health care encounters with an associated *International Classification of Diseases, Ninth Revision (ICD-9) *or *ICD-10* code for falls or fall-related injuries (eTable 2 in Supplement 2).

### Independent Variables

The primary independent variable was clinic type (intervention or control). Covariates included baseline medication exposure (average daily MMEs or DMEs ordered during the 1-year preintervention period). Demographic characteristics gathered from the EHR included age (65-69, 70-79, 80-89, or ≥90 years), sex, race and ethnicity (African American, Hispanic, White, or other, including Asian and multiracial), and Medicaid enrollment. Data on race were collected because White women have a higher incidence of falls. We included self-reported falls in the prior year and visits for fall-related injuries. Self-reported falls were extracted via a discrete data field in the EHR, which documents whether patients have fallen in the last year, and visits for fall-related injuries were identified based on *ICD-10* codes. Relevant comorbid conditions were identified based on *ICD-10* codes (major depression, posttraumatic stress disorder, anxiety, alcohol-related disorders, opioid use disorder, insomnia, and chronic pain).^29,30^ The study also considered potential coprescribing of central nervous system (CNS)–acting medications using the AGS Beers Criteria^31^ and the Centers for Disease Control and Prevention’s STEADI-Rx tool.^32^ We evaluated the presence of CNS polypharmacy, defined as receiving 2 or more CNS-active medications in addition to opioids and/or benzodiazepines.

### Statistical Analysis

Analyses followed an intention-to-treat approach. Data analysis was performed using SAS, version 9.4 (SAS Institute Inc) in November 2022. We used analysis of covariance to evaluate the difference in mean medication exposure during the 1-year postintervention period. We used generalized linear models (GLMs) to evaluate the association of the intervention with log-transformed average daily MMEs and DMEs in separate models. The GLM was fitted using iteratively reweighted least squares (ie, generalized estimating equations) with exchangeable working correlation matrix and model-based clustered robust SEs. Three models were conducted to adjust for potential confounders: model 1 was unadjusted, model 2 was adjusted for baseline medication exposures MMEs and DMEs, and model 3 was adjusted for baseline medication exposures and demographic characteristics (eg, age, sex, race, ethnicity, and Medicaid use). Model-based SEs, accounting for potential clinic clustering were used, reporting effect size and 95% CIs and representing percent difference in average daily MMEs or DMEs among patients seen in intervention vs control clinics.

Logistic regression assessed the odds of medication discontinuation and incident falls in the postintervention period among clinics. We used the 3 models described above for class to adjust for potential confounders. We used model-based SEs to address potential clinic-level clustering. Results, presented as odds ratios (ORs) with 95% CIs, indicate the odds of discontinuation or incident fall among patients seen in intervention compared with control clinics. Two-sided *P* < .05 was considered statistically significant.

We conducted several sensitivity analyses based on a priori hypotheses and a preliminary examination of interaction terms between the primary independent variable and demographic characteristics. A priori hypotheses considered intervention effectiveness in relation to baseline prescription doses, categorizing high-level prescribing as average daily MMEs greater than 50 and average daily DMEs greater than 10.^8,33^

## Results

### Sample Characteristics

A total of 2075 patients (mean [SD] age, 74.6 [0.13 for opioid group and 0.15 for benzodiazepine group] years; 1429 [68.9%] female, 639 [30.8%] male, and 7 [0.3%] unknown sex; opioid group: 131 [13.6%] African American, 5 [<.05%] Hispanic, 819 [84.8%] White, and 11 [<0.5%] other race [Asian or multiracial]; benzodiazepine group: 64 [5.7%] African American, 9 [<0.5%] Hispanic, 1026 [91.9%] White, and 17 [<0.5%] other race [Asian or multiracial]) met criteria for long-term opioid or benzodiazepine use. A greater number of patients taking benzodiazepines were women and White compared with those taking opioids, as reported elsewhere.^34^ Among patients with long-term opioid use, 312 (32.5%) reported a fall in the past year, with 103 (10.7%) showing evidence of a fall-related injury visit (Table 1). A total of 920 (95.7%) had a chronic pain diagnosis. Fewer patients receiving benzodiazepines reported a fall in the past year (286 [25.8%]) or had evidence of a visit for a fall-related injury (87 [7.9%]). Prevalent indications for treatment were anxiety (496 [44.8%]) and insomnia (281 [25.4%]). Coprescribing of both opioids and benzodiazepines was present in 150 older adults (15.6% of the opioid group and 13.6% of the benzodiazepine group). Additionally, 441 patients receiving opioids (45.9%) and 469 receiving benzodiazepine (42.3%) had evidence of CNS polypharmacy.Baseline Medication Exposures

**Table 1.** Baseline Characteristics of Older Adults (≥65 Years) From Enrolled Primary Care Practices and Prescribed Opioid and/or Benzodiazepine Use

Characteristic	No. (%) of patients^e^					
	Long-term opioid use			Long-term benzodiazepine use		
	Intervention (n = 574)	Control (n = 387)	Standardized mean difference	Intervention (n = 536)	Control (n = 571)	Standardized mean difference
Demographics						
Age group, y						
65-69	174 (30.3)	100 (25.8)	0.13	167 (31.2)	156 (27.3)	0.15
70-79	248 (43.2)	191 (49.4)		245 (45.7)	292 (51.1)	
80-89	128 (22.3)	83 (21.5)		108 (20.2)	115 (20.1)	
≥90	24 (4.2)	13 (3.4)		16 (3.0)	8 (1.4)	
Sex						
Female	367 (63.9)	255 (65.9)	0.04	378 (70.5)	429 (75.1)	0.10
Male	207 (36.1)	132 (34.1)		158 (29.5)	142 (24.9)	
Race and ethnicity						
African American	78 (13.6)	53 (13.7)	0.06	37 (6.9)	27 (4.7)	0.09
Hispanic	4 (0.7)	1 (0.2)		3 (0.6)	6 (1.1)	
White	488 (85.0)	331 (85.5)		491 (91.6)	535 (93.7)	
Other^a^	8 (1.4)	3 (0.8)		8 (1.5)	9 (1.6)	
Medicaid enrollment	124 (21.6)	30 (7.7)	0.39	81 (15.1)	33 (5.7)	0.31
Clinical characteristics						
Self-reported fall in prior year	187 (32.6)	125 (32.3)	<0.01	157 (29.3)	129 (22.6)	0.15
Preencounter for fall-related injury^b^	63 (11.0%)	40 (10.3)	0.06	50 (9.3)	37 (6.6)	0.16
Major depression^c^	165 (28.8)	106 (27.4)	0.03	167 (31.2)	162 (28.4)	0.06
PTSD^c^	4 (0.7)	5 (1.3)	0.06	8 (1.5)	7 (1.2)	0.02
Other anxiety^c^	104 (18.1)	61 (15.8)	0.06	262 (48.9)	234 (41.0)	0.16
Alcohol-related disorder^c^	11 (1.9)	8 (2.1)	0.01	9 (1.7)	13 (2.3)	0.04
Opioid-use disorder^c^	6 (1.1)	5 (1.3)	0.02	8 (1.5)	4 (0.7)	0.08
Insomnia^c^	73 (12.7)	76 (19.6)	0.18	121 (22.6)	160 (28.0)	0.12
Chronic pain^c^	549 (95.6)	371 (95.9)	0.01	418 (78.0)	447 (78.3)	<0.01
Other chronic pain^c^	295 (51.4)	165 (42.6)	0.17	159 (29.7)	111 (19.4)	0.23
Medication use						
CNS polypharmacy^d^	274 (47.7)	167 (43.2)	0.09	237 (44.2)	232 (40.6)	0.07
Average (SD) daily MMEs or DMEs before the intervention^f^	26.9 (54.2)	18.7 (33.4)	0.18	8.8 (10.4)	6.6 (8.2)	0.23
Median (IQR) daily MMEs or DMEs before the intervention^f^	11.3 (5.5-29.1)	9.2 (4.3-19.0)	0.16	5.0 (2.3-10.3)	4.3 (1.7-9.3)	0.14
High-level exposure (>50 MMEs or >10 DMEs)	72 (12.5)	28 (7.2)	0.17	137 (25.6)	92 (16.1)	0.23
Receiving both opioids and benzodiazepines	91 (15.9)	59 (15.3)	0.02	91 (17.0)	59 (10.3)	0.19

Abbreviations: CNS, central nervous system; DMEs, diazepam milligram equivalents; MMEs, morphine milligram equivalents; PTSD, posttraumatic stress disorder.

^a^ Asian or multiracial.

^b^ *International Statistical Classification of Diseases and Related Health Problems, Tenth Revision (ICD-10) *used to identify fall- or fracture-related medical encounter using electronic health records (see eTable 2 in Supplement 2).

^c^ *ICD-10* used for the condition using electronic health records (see eTable 2 in Supplement 2).

^d^ Receiving 2 or more CNS active medications in addition to opioids and/or benzodiazepines.

^e^ Unless otherwise indicated.

^f^ Opioid use is measured in MMEs and benzodiazepine use in DMEs.

**Figure 2.**
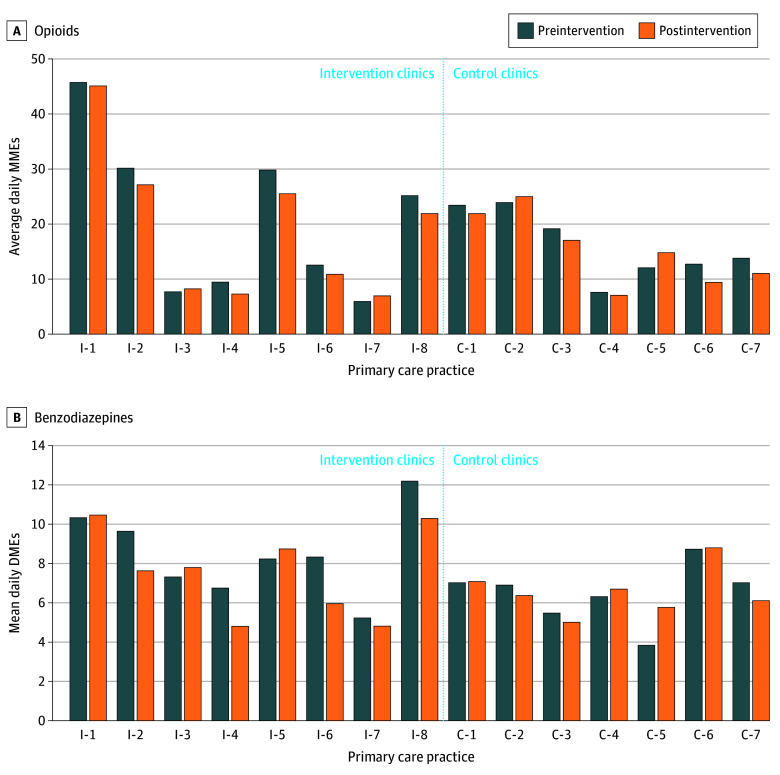
Clinic-Level Prescribing of Opioids and Benzodiazepines Before and After Intervention Among Older Adults (≥65 Years) From Enrolled Primary Care Practices C indicates control clinic; DMEs, diazepam milligram equivalents; I, intervention clinic; MMEs, morphine milligram equivalents.

Baseline average daily MMEs was 23.6 for opioids, with 100 older patients (10.4%) being prescribed more than 50 MMEs daily (Table 1). Average daily opioid use was greater in intervention clinics compared with control clinics (26.9 vs 18.7 average daily MMEs). The average daily MMEs prescribed to patients at baseline in each clinic ranged from 5.9 to 45.7 (Figure 2A). Among opioids, tramadol (471 [49.0%]), hydrocodone (363 [37.7%]), and oxycodone (327 [34.0%]) were the most ordered medications (eTable 3 in Supplement 2).

Baseline average daily DMEs was 7.7 for benzodiazepines, with older patients being prescribed more than 10 DMEs daily. Average daily benzodiazepine use was also greater in intervention clinics compared with control clinics (8.8 vs 6.6 average daily DMEs). The range of average daily DMEs prescribed to patients at baseline varied from 3.8 to 12.4 (Figure 2B). Among benzodiazepines, the most ordered medications were alprazolam (427 [38.6%]), clonazepam (277 [25.0%]), and lorazepam (260 [23.5%]) (eTable 3 in Supplement 2).

### Pharmacist Recommendations

Of the 574 patients with long-term opioid use, recommendations for a taper were provided for 408 (71.1%). Of these, 241 (59.1%) were accepted by primary care practitioners. Of the remaining patients, 73 (12.7%) were deemed ineligible for a taper recommendation by the pharmacist, and the remaining 93 patients (16.2%) canceled or did not show to their appointment after the pharmacist reviewed the patient’s information.

Of the 536 patients with long-term benzodiazepine use, recommendations for a taper were provided for 383 (71.5%). Of these, 260 (67.9%) were accepted by primary care practitioners. For the remaining patients, 71 (13.2%) were deemed ineligible for a taper by the pharmacist, and the remaining 82 (15.3%) either canceled or did not show to their appointment after the pharmacist reviewed the patient’s information.

### Assessment of Outcomes

In the 1-year postintervention period, average daily MMEs decreased in both the intervention (26.9 vs 24.5, 8.8% reduction) and control (18.6 vs 17.6, 5.4% reduction) clinics (Table 2). Reductions varied widely by clinic, with the largest reduction in average daily MMEs for an intervention clinic being 34.7% (Figure 2; eTable 4 in Supplement 2).

**Table 2.** Medication Exposures Before and After Intervention Among Older Adults (≥65 Years) From Enrolled Primary Care Practices and Prescribed Opioid and/or Benzodiazepine Use

Exposure	Long-term opioid use			Long-term benzodiazepine use	
	Intervention (n = 574)		Control (n = 387)	Intervention (n = 536)	Control (n = 571)
**Preintervention exposures**					
Average (SD) daily exposure^c^	26.9 (54.2)		18.7 (33.4)	8.8 (10.4)	6.6 (8.2)
Median (IQR) daily exposure^c^	11.3 (5.5-29.1)		9.2 (4.3-19.0)	5.0 (2.3-10.3)	4.3 (1.7-9.3)
High-level exposure (>50 MMEs or >10 DMEs), No. (%)	72 (12.5)		28 (7.2)	137 (25.6)	92 (16.1)
Receiving both opioids and benzodiazepines, No. (%)	91 (15.9)		59 (15.3)	91 (17.0)	59 (10.3)
**Postintervention exposures**					
Average (SD) daily exposure^c^	24.5 (50.4)		17.6 (32.0)	7.8 (10.1)	6.5 (8.3)
Median (IQR) daily exposure^c^	9.9 (4.5-27.1)		9.1 (4.1-17.5)	3.7 (1.5-10.0)	4.3 (1.6-9.0)
High-level exposure (>50 MMEs or >10 DMEs), No. (%)	71 (12.4)		27 (7.0)	127 (23.7)	87 (15.2)
**Intervention effect^a^**					
Unadjusted effect	0.30 (−0.15 to 0.74)	NA		0.18 (0.02 to 0.35)	NA
*P* value	.19	NA		.03	NA
Adjusted for baseline MMEs only	0.043 (−0.12 to 0.21)	NA		−0.12 (−0.25 to 0.01)	NA
*P* value	.61	NA		.08	NA
Primary analytic model					
Adjusted for baseline MMEs and demographics^b^	0.032 (−0.14 to 0.20)	NA		−0.12 (−0.25 to 0.01)	NA
*P* value	.71	NA		.07	NA

Abbreviations: DMEs, diazepam milligram equivalents; MMEs, morphine milligram equivalents.

^a^ β coefficient × 100 can be interpreted as the percent difference in MMEs or DMEs prescribed in intervention clinics vs control clinics 1 year after the index visit. For larger magnitude values (eg, >0.10), this approximation diverges from this interpretation and indicates larger change. The correct calculation would be exp(β-coefficient).

^b^ Age, sex, race, ethnicity, and Medicaid.

^c^ Opioid use is measured in MMEs and benzodiazepine use in DMEs.

In GLMs, which represent the percentage difference in average daily MMEs among patients in intervention vs control clinics, we found no significant differences (model 1 effect, 0.30; 95% CI, −0.15 to 0.74; model 2 effect, 0.04; 95% CI, −0.12 to 0.21; model 3 effect, 0.03; 95% CI, −0.14 to 0.20) (Table 2). Although the proportion of patients who discontinued opioids was 21.4% in intervention clinics and 19.9% in control clinicals, this difference was not statistically significant (OR, 1.20; 95% CI, 0.85-1.71) (eTable 5 in Supplement 2) Additionally, there was no difference in the likelihood of incident falls between patients taking opioids in control and intervention clinics (OR, 1.36; 95% CI, 0.94-1.96) (eTable 5 in Supplement 2).

Mean (SD) daily MMEs decreased by 8.8% in intervention clinics (26.9 [54.2] to 24.5 [50.4]) and 5.4% in controls (18.7 [33.4] to 17.6 [32.0]; *P* = .71); mean (SD) DMEs decreased by 11.4% (8.8 [10.4] to 7.8 [10.1]) and 1.5% (6.6 [8.2] to 6.5 [8.3]), respectively (*P* = .07) (Table 2). The largest reduction in average daily DMEs for an intervention clinic was 28.8% (Figure 2; eTable 6 in Supplement 2). GLMs again showed no significant differences (model 1 effect, 0.18; 95% CI, 0.02-0.35; model 2 effect, −0.12; 95% CI, −0.25 to 0.01; model 3 effect, −0.12; 95% CI, −0.25 to 0.01) (Table 2). The proportion of patients who discontinued benzodiazepines was 22.0% in intervention clinics and 18.4% in control clinics,, this difference was not statistically significant (OR, 1.41; 95% CI, 0.94-2.03) (eTable 5 in Supplement 2). Lastly, fall risk was unchanged (OR, 1.19; 95% CI, 0.67-2.10) (eTable 5 in Supplement 2).

### Sensitivity and Subgroup Analyses

Results were unchanged for patients taking opioids (eTable 7 in Supplement 2). Among patients taking benzodiazepines with less than 10 daily DMEs, the intervention significantly reduced DMEs (effect size, −0.22; 95% CI, −0.36 to −0.08; *P* = .002) (eTable 8 in Supplement 2). The findings of all other sensitivity analyses were not significant (eTable 9 in Supplement 2).

## Discussion

To our knowledge, this is the first large cluster randomized trial to evaluate deprescribing of both opioids and benzodiazepines in older adults through a consultant pharmacist model.^23^ Although reductions occurred in both groups, the intervention did not significantly reduce prescribing or falls at 1 year. Still, pharmacist recommendations were feasible to implement and accepted more often than rejected, indicating general practitioner receptiveness. Longer, more intensive, or multimodel interventions may be necessary to produce stronger deprescribing outcomes.

Although most recommendations were accepted, the 1-year period was too short to achieve statistically significant dose reductions; however, one could argue that the dose reductions may have been clinically meaningful. For clinically meaningful changes in opioid or benzodiazepine use, especially for patients with a long-standing history of use and higher doses, tapering may take years. Many prescribers expressed that they liked the consultant pharmacist model intervention.^35^ In fact, patients who were prescribed less than 10 DMEs per day demonstrated a statistically significant reduction in average daily DMEs. This finding suggests that patients with lower exposures may be more suitable to a low-touch deprescribing intervention, whereas patients receiving higher doses may require sustained interventions, adjunctive treatments, or more direct pharmacist to patient engagement to facilitate deprescribing.

Pharmacists have been established as valuable members of interprofessional health care teams^36^; however, embedding them in teams can be cost prohibitive.^37^ Our study’s intervention, relying on a centralized team of consultant pharmacists, aimed to minimize workflow disruptions and enhance efficiency. However, we recognize that practitioners may struggle to prioritize deprescribing discussions over other competing clinical issues, even with tailored recommendations. One of the shortcomings of this approach is the lack of direct engagement of pharmacists with both patients and prescribers, which may have contributed partially to the lack of significant impact.

As mentioned previously, the intervention period was relatively short. A 2-year study demonstrated that a pharmacist-led benzodiazepine-tapering clinic was an effective way to engage motivated patients.^38^ One of the most successful large-scale interventions to reduce benzodiazepine use has been the EMPOWER trial,^39^ which used mailed brochures to motivate patients to discuss benzodiazepine use with their prescribers and reduce use. Future studies should seek to evaluate whether lower-resource interventions, such as direct-to-consumer education combined with a consultant pharmacist intervention, would have a synergistic effect on reducing high-risk medication use for older adults. Additionally, addressing fall risk in older adults requires a comprehensive multifactorial approach considering other factors, such as comorbidities, environmental hazards, and physical limitations.

### Strengths and Limitations

Strengths of this study include the cluster randomized design, large sample size, multiclinic participation, and structured EHR integration. This study also has limitations, including baseline imbalances among clinics, reliance of prescription order rather than dispensing data, and potential under capture of falls using *ICD-9* and *ICD-10* codes. The study may also have been underpowered for the small differences observed. Nevertheless, the model shows promise, especially for rural clinics, where consultant pharmacist support could help address resource limitations.

## Conclusions

In this large cluster randomized trial, a centralized consultant pharmacist intervention did not significantly reduce opioid or benzodiazepine prescribing or falls among older adults during a 1-year period, although modest decreases were observed in both intervention and control clinics. The intervention was feasible to implement, integrated smoothly into the EHR, and led to high acceptance of pharmacist recommendations by primary care practitioners. Ultimately, this demonstrated the practicality of this deprescribing support model. This study’s findings suggest that low-intensity, remote pharmacist interventions may offer limited impact for patients with long-standing or high-dose opioid and benzodiazepine use but may be more effective for those with lower baseline benzodiazepine exposures. Future work should evaluate whether augmenting consultant pharmacist services with direct patient engagement, multimodal clinical support, and longer intervention periods can yield more substantial medication reductions and fall prevention benefits. As health systems seek scalable strategies to reduce high-risk medication use in aging populations, consultant pharmacist models, particularly in resource-limited or rural settings, remain a promising foundation for more comprehensive deprescribing approaches.

## Notes

Supplement 1.
Trial Protocol

Supplement 2. **eTable 1. **Clinic Characteristics **eTable 2. **List of Relevant Indications for Opioid and Benzodiazepine (BZD) Use **eTable 3. **Prescribing Data by Generic Drug Name Among Older Adults (65+) From Enrolled Primary Care Practices **eTable 4. **Average Daily Morphine Milligram Equivalents by Clinic (Pre- and Post-intervention) **eTable 5. **Intervention Effectiveness for Secondary Outcomes **eTable 6. **Average Daily Diazepam Milligram Equivalents by Clinic (Pre- and Post-intervention) **eTable 7. **Effectiveness of Opioid Deprescribing Intervention on Average Daily Morphine Milligram Equivalents - High vs. Low Baseline Exposure **eTable 8. **Effectiveness Benzodiazepine Deprescribing Intervention on Average Daily Diazepam Milligram Equivalents – High vs. Low Baseline Exposure **eTable 9. **Effectiveness of Opioid Deprescribing Intervention on Average Daily Morphine Milligram Equivalents – Older or Younger Than 80 Years of Age

Supplement 3.
Data Sharing Statement
