# Supplementary material for: A Pharmacist Consultant Service for Deprescribing Opioids and Benzodiazepines in Older Adults: A Cluster Randomized Trial
Source: JAMA Netw Open. 2026 Feb 26;9(2):e2560581. doi: 10.1001/jamanetworkopen.2025.60581 (PMC12947021; doi:10.1001/jamanetworkopen.2025.60581)
Supplement: Supplement 1. — Trial Protocol [file jamanetwopen-e2560581-s001.pdf]

Complete Title: Implementation of a Deprescribing Medication Program to Evaluate Falls in Older Adults

Short Title: Deprescribing Medications to Prevent Falls

Sponsor: Centers for Disease Control and Prevention

Protocol Date: 5/22/2019

Centers for Disease Control and Prevention
2920 Brandywine Road
Atlanta, GA 30341

PROTOCOL TITLE: Implementation of a Deprescribing Medication Program to Evaluate Falls in Older Adults

Short Title: Deprescribing Medications to Prevent Falls

Lead Investigator: Stefanie Ferreri, PharmD

University of North Carolina at Chapel Hill

Protocol Version: 1.0

Version Date: May 22, 2019

I confirm that I have read this protocol and understand it.

Principal Investigator Name: Stefanie Ferreri

Principal Investigator Signature: 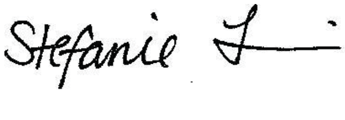

Date: 5/22/2019

TABLE OF CONTENTS

TABLE OF CONTENTS 3

ABBREVIATIONS AND DEFINITIONS OF TERMS 4

PROTOCOL SYNOPSIS 5

2 STUDY OBJECTIVE 9
3 INVESTIGATIONAL PLAN 10
4 STUDY PROCEDURES 12
5 SCREENING AND MONITORING EVALUATIONS AND MEASUREMENTS 14
6 STATISTICAL CONSIDERATION 15
7 SAFETY MANAGEMENT 17
8 DATA COLLECTION AND MANAGEMENT 18
9 RECRUITMENT STRATEGY 19
10 CONSENT PROCESS 19
11 PLANS FOR PUBLICATION 19
12 REFERENCES 20
13 APPENDIX ..... **Error! Bookmark not defined.**

ABBREVIATIONS AND DEFINITIONS OF TERMS

Abbreviation	Definition
BZD	Benzodiazepine
CDWH	Carolina Data Warehouse for Health
CDC	Centers for Disease Control and Prevention
CSV	Comma-separated values
DME	Diazepam milligram equivalents
EHR	Electronic health record
GEE	Generalized estimating equations
HCP	Healthcare provider
ITS	Information Technology Services
MME	Morphine milligram equivalents
STEADI	Stop Elderly Accidents, Deaths and Injuries

PROTOCOL SYNOPSIS

**Study Title**	Implementation of a Deprescribing Medication Program to Evaluate Falls in Older Adults
**Funder**	Centers for Disease Control and Prevention
**Study Rationale**	Falls among older adults in the US account for 2.8 million injuries, 800,000 hospitalizations, and 28,000 deaths per year.[1] US medical costs associated with falls now total approximately $50 billion.[2] More than 150 randomized trials document successful falls interventions.[3] Observational studies show consistent associations between falls and both opioids and benzodiazepines (BZD). Thus, it is widely accepted that fall-related morbidity and mortality could be reduced if systems were in place to identify and intervene in falls risk. Unfortunately, most physicians do not routinely screen older patients for falls[4] and with the shortage of physicians going into primary care, it is unlikely that screening will improve. Therefore, an educational intervention supporting screening for risk of falls and deprescribing of opioids and benzodiazepines may have a meaningful impact on the fall risk in an elderly population.
**Study Objective(s)**	**AIM 1:** Use EHR data to inform outpatient clinics of older adults who are at high risk of falls due to the chronic use of opioids and/or BZDs. Once alerted, clinics will use a team-based approach to screen and plan falls interventions focused on deprescribing.<br>**AIM 2:** Develop and implement a proactive deprescribing medication guidance document with supporting resources (toolkit) for use with older adults on opioids and/or BZDs.<br>**AIM 3:** Evaluate (a) the utilization of the deprescribing toolkit in practice; (b) the satisfaction of clinic teams with the toolkit; (c) the impact of the toolkit on opioid and BZD exposure (primary outcome variable); (d) the rate of falls (secondary outcome variable).
**Study Design**	This **mixed method study** incorporates two distinct study designs. **Focus groups** will be used to inform the development and refinement of a deprescribing guidance document with supporting resources. This document will define the provider-focused educational intervention which will be tested using a **cluster-randomized design**. Eligible providers will be randomly assigned to intervention and control groups. Impact of the educational intervention on medication use and falls will be assessed using an **longitudinal pre-treatment post-treatment design**, with patients receiving care from control clinics serving as a quasi-equivalent comparison group. Data from clinics will also be supplemented by a **web-based survey**, which will be given twice: prior to the start of the study and after its completion.
**Subject Population key criteria for Inclusion and Exclusion:**	**Inclusion Criteria:** To be eligible to participate in the study, clinics need to be affiliated with UNC HealthCare and have a patient population that includes patients 65 years of age or older. The educational intervention will target patients at least 65 years old and taking at least one opioid or benzodiazepine medication listed

	in the design section for > 6 weeks, indicating they are at high risk for falls.<br><br>To be eligible to participate in the patient focus group, participants need to be a patient in one of the intervention group clinics, 65 years of age and older, and taking at least one opioid or benzodiazepine chronically.<br><br>**Exclusion Criteria**<br>Clinics that do not provide primary care services will be excluded from participating in the study.<br><br>Patients who exhibit signs of cognitive impairment or speech/hearing deficits that make obtaining informed consent and completing data collection activities difficult; patients undergoing active cancer treatment, receiving hospice care, or living in a skilled nursing facility; and non-English speaking patients will be excluded from participating in the patient focus group. Participants who do not wish to be audio-recorded during focus group sessions will also be excluded.<br><br>Patients who exhibit signs of cognitive impairment; are non-English speaking; or are undergoing active cancer treatment, receiving hospice care, or living in a skilled nursing facility will be excluded from the deprescribing intervention.
**Number Of Subjects**	**Focus groups:** 12 healthcare professionals and 12 patients.<br>**Web-based survey**: 120 healthcare professionals<br>**Cluster-randomized trial:** Total number of clinics (intervention & control): 20; Total number of patients (intervention and control): 3,000
**Study Duration**	**Focus groups:** two focus groups (120 minutes each)<br>**Web-based survey:** 1 survey, given twice: pre-study and post-study<br>**Cluster-randomized trial:** 1 year<br>The entire study is expected to last 4 years
**Study Phases**<br>**Screening**<br>**Observation**	**Focus groups:** Participants will sign a consent form prior to the start of the focus group session.<br>**Web-based surveys**: The surveys will include a written statement stating that their participation in the survey is voluntary and by completing the survey they are consenting to participate.<br>**Cluster-Randomized Trial:** Each clinic's 'study champion' will sign a consent form prior to the clinic's participation.
**Safety Evaluations**	The risk from focus groups and web-based surveys is expected to be minimal. To monitor safety from the educational intervention, we

	will include specific monitoring criteria in the educational intervention itself (e.g., monthly follow-up with clinic champions about the study), with specific instructions to alert study staff if any adverse effects of deprescribing are observed. Additionally, a study team member will reach out to all intervention clinics monthly to ask about potential safety risks related to deprescribing.
**Statistical And Analytic Plan**	**Focus Groups:** Thematic analysis<br>**Cluster-Randomized Trial:**<br>• Primary: Longitudinal pre-post analysis to detect impact of program on dose of opioid and opioid discontinuation<br>• Secondary: Rate of falls<br>**Web-based surveys:** Descriptive statistics
**DATA AND SAFETY MONITORING PLAN**	This study will not have a separate data safety monitoring board. This decision was made because the intervention is usual care that is provided from the clinics. If there are incidents related to deprescribing the clinics will follow their protocols and refer patients to urgent care, the emergency room or have an on-call provider call the patient back. This is usual care. Information about adverse effects will be reported to the PI during the monthly check in. Side effects from discontinuation are a known risk from deprescribing opioids and BZDs, therefore they will be dealt with through the typical care system, which includes evening and weekend call hours for primary care clinics, access to the emergency department, and other standard care options for patients. The PI will follow up with the prescriber once notified to ensure that the usual care for handling side effects from discontinuation were followed.

1 BACKGROUND AND RATIONALE

1.1 Introduction

Falls among older adults in the US account for 2.8 million injuries, 800,000 hospitalizations, and 28,000 deaths per year.[1] US medical costs associated with falls now total approximately $50 billion.[2] More than 150 randomized trials document successful falls interventions.[3] Observational studies show consistent associations between falls and certain medications. Thus, it is widely accepted that fall-related morbidity and mortality could be reduced if systems were in place to identify and intervene in falls risk. Unfortunately, most physicians do not routinely screen older patients for falls[4] and with the shortage of physicians going into primary care, it is unlikely that screening will improve.

The University of North Carolina at Chapel Hill (UNC) School of Pharmacy pioneered an approach using community-based pharmacists to screen and identify older adults at risk.[5-7] In our study, pharmacists identified 186 patients who were on a high-risk medication. Pharmacists then conducted a medication review using a clinical algorithm to guide the decision-making process. Twenty-five percent of the pharmacists' recommended medication changes were accepted by physicians. In our current, follow-up, CDC-funded study, the UNC Schools of Pharmacy and Medicine are using an enhanced service network of 65 pharmacies to mitigate the barriers faced by community pharmacists conducting medication reviews. As of March 1, 2018, 70% of qualified patients were screened by pharmacists and 56% received a medication review with recommendations for lower risk drugs. Of these, 35% of have been accepted by prescribers. These studies show that other members of a healthcare team,

specifically, pharmacists, can accurately identify older adults at risk for falls and communicate recommendations for medication changes.

1.2 Potential Risks and Benefits

There are three primary methods used for this study: focus groups, web-based surveys, and a cluster-randomized trial. The risks from focus groups and web-based surveys are minimal and primarily involve the possibility of loss of confidentiality and participants feeling uncomfortable.

The primary risk for the cluster-randomized study is potential withdrawal symptoms from discontinuation of opioids and BZDs. This risk is part of the standard of care associated with opioid and BZD management and is no greater than the normal risk experienced with standard patient care. The goal of this study is to better inform clinicians as to the fall-related risks associated with elderly patients' use of opioids and BZDs as well as best practices for tapering patients off opioids and BZDs without excess risk. If a clinician deems the falls-related benefit from reducing or stopping an opioid or BZD outweighs the risk associated with withdrawal, they can work with a patient to reduce or stop their medication. As such, potential benefits to the patient include a reduced risk of falls, which are associated with substantial morbidity and mortality in older adults.

1.3 Relevant Literature and Data

Prevalence and Consequences of Falls among Older Adults. Falls are the leading cause of unintentional fatal and nonfatal injury among adults age 65 and older in the US.[8,9] According to the latest figures, one in four older adults fall each year.[9] Outcomes associated with falls and fall-related injuries include: functional impairment, loss of independence, emotional distress, and increased use of health and social services.[10-13] Fractures, particularly hip fractures, are the most common and expensive nonfatal fall-related injury, accounting for about one-third of fall injuries and 60% of associated medical costs.[14] Reducing the burden of falls among older adults is a significant public health challenge.

Medication Management and Falls. Past research demonstrates that individuals who use multiple medications are at increased risk for falls.[15,16] In observational studies, consistent associations have been found between the use of high-risk medications such as opioids and benzodiazepines (BZD) medications and an increased risk of falls.[15,17-20] Prior research has demonstrated that pharmacists can significantly reduce or eliminate medications that place patients at high risk of falls by performing medication reviews.[21-23] However, researchers conclude that medication reviews alone do not lead to a reduction in overall falls.[5,24] In our experience, opioid and BZD classes of drugs are the most difficult to manage in patients. Therefore, research focusing on medication management of opioids and BZDs is necessary to reduce falls among older adults.

A literature review of 239 peer reviewed articles revealed several studies citing the link between high-risk medications and falls and subsequently calling for deprescribing algorithms and education.[25-27] The first tool for deprescribing these medications was the Beers Criteria published in 1991. Mark Beers and a panel of experts created a list of potentially inappropriate medications for older adults. While the use of many Beers list medications declined, implementing the list as a stand-alone resource, without educational support, lead to an overly rigid application, essentially demonizing even the appropriate use of these medications in some settings.[28]

In 2011, the Canadian guideline for safe and effective use of opioids for chronic non-cancer pain gave specific deprescribing advice to family physicians.[29] An implementation of a deprescribing protocol in Japan resulted in pharmacists recommending medication changes 48% of the time (310 out of 651 medications reviewed). Ultimately, 292 medications were changed.[30] This matches the experience of a previous randomized controlled trial conducted by one of the co-PIs. In this trial, 186 patients with a

history of falls who were taking high-risk medications received either a face-to-face medication consultation at a community pharmacy or usual care. Those in the treatment group were more likely to discontinue the use of a high-risk medication or have their dosages reduced.[5]

Over time, researchers have added patient education and non-pharmacological strategies as active interventions in deprescribing protocols so that clinicians have more than just a list of prohibited medications to work with.[22] A 2010 study of 591 community dwelling older adults prescribed BZDs or a related drug showed that instructing patients on how to discontinue these medications and why they should do so prompted a 35% reduction in use, while the control group, which received no such instructions, increased their use by 4%.[31] An inpatient study in Ireland found only 20% of falls risk medications were suitable for change. Given that medication reviews were time consuming, the researchers recommended targeting certain medications.[32]

Keeping patients from resuming the use of BZDs or opioids over the long term is also a challenge. A meta-analysis of 18 randomized trials found moderate success in deprescribing psychotropic medications. Four of these projects reported falls outcomes. One of the studies reported a 66% reduction in falls, but 47% of patients resumed the use of the medications; therefore, clinicians need a strategy of long-term interventions.[33]

The literature shows progression over time in our awareness of the role of medications in falls and an increasing sophistication of interventions, including successful interventions in pharmacies. Policy makers and payers are starting to support these efforts. The Centers for Medicare and Medicaid Services regulations including Medicare Part D now require medication reduction strategies and numerous quality improvement projects focus on reducing high-risk medications to prevent falls.[34]

Engaging the Entire Health Care Management Team in Falls Prevention for Older Adults. The American Geriatrics Society/British Geriatrics Society Clinical Practice Guideline for Prevention of Falls in Older Persons recommends that healthcare providers (HCPs) ask all older adults about falls, their frequency of falling, and difficulties in gait or balance on an annual basis.[35] However, this screening occurs infrequently in clinical practice. For example, in one study, only 25% of older adult patients reported they had been asked about falls annually and only 48% reported they had ever been asked about falls.[36] Another study, based on a nationally-representative sample of Medicare beneficiaries, found that less than 30% of the individuals who had experienced a fall within the previous year had received counseling about fall prevention from their health care provider.[37] We could only find one study that engaged the entire health care team in reducing the risk of falls.[38] Engaging the entire health care team may help patients adopt and maintain behaviors that reduce falls risk especially as it relates to opioid and BZD deprescribing. Regarding deprescribing, only a few studies focus on a patient centered approach including education and cognitive behavioral therapy in addition to deprescribing.[39-41] This study will use a team-based approach to screen patients and de-prescribe opioids and BZDs for patients at risk for falls in the outpatient setting.

Impact of Proposed Study. This will be one of the first cluster-randomized studies targeting deprescribing of opioids and benzodiazepines in outpatient clinics with the intent to reduce falls in older adults**.** Implementing a multidisciplinary deprescribing educational toolkit within 10 outpatient clinics will promote replication and expansion, significantly reducing falls in the target population. Ultimately, this toolkit can be distributed to outpatient clinics nationally.

2 STUDY OBJECTIVE

AIM 1: Use EHR data to inform primary care outpatient clinics of older adults who are at high risk of falls due to the chronic use of opioids and/or BZDs. Once alerted, clinics will use a team-based approach to screen and plan falls interventions focused on deprescribing.

AIM 2: Develop and implement a proactive deprescribing medication guidance document with supporting resources (toolkit) for older adults on opioids and/or BZDs.

AIM 3: Evaluate (a) the utilization of the deprescribing toolkit in practice; (b) the satisfaction of clinic teams with the toolkit; (c) the impact of the toolkit on opioid and BZD exposure (primary outcome variable); (d) the rate of falls (secondary outcome variable).

2.1 Primary Objective

Focus Groups: To inform the development and refinement of the opioid and benzodiazepine deprescribing toolkit (guidance document with supporting resources) and associated algorithm.

Web-Based Survey: To capture provider-reported barriers to and facilitators for the provision of deprescribing services as well as provider-reported demographics.

Cluster-Randomized Trial: To reduce opioid and BZD exposure, as measured by dose and discontinuation rate.

2.2 Secondary Objective

Cluster-Randomized Trial: To evaluate the rate of falls.

3 INVESTIGATIONAL PLAN (brief overview)

3.1 Study Design

This study consists of three distinct sets of methodologies: focus groups, a web-based survey, and a cluster-randomized trial.

3.1.1 Focus Groups:

The patient focus group will be conducted in-person. The patient focus group participants will be asked open-ended questions around the following themes: (1) their attitudes toward use of opioids or BZDs to manage chronic pain or sleep respectively; (2) what level of concern do they have, if any, with respect to dependence on their medications; (3) what level of concern, if any, do they have with respect to the risks of overdose; (4) what experiences have they had with withdrawal attempts or symptoms; (5) what other therapies are they are aware of; (6) what other therapeutic approaches have they tried/or would they try; (7) what barriers, if any have they experienced in using alternative therapies; and (8) what knowledge do they have about the correlation between their medications and the risk of falls. Audio recordings will be transcribed and thematically coded. To ensure anonymity, names will not be recorded, and tapes will be destroyed immediately after transcription. Study participants may receive nominal compensation as well as transportation reimbursement. Three independent coders will review the tapes. During coding, three coders will 1) study the textual material; 2) pinpoint recurring quotes; 3) examine them vis-à-vis related or contradictory quotes also in the text; and 4) organize them into major themes. As part of axial coding, the coders can compare notes and negotiate themes that provide the richest understanding of the study participants' knowledge and experiences.

The provider focus group will also be conducted in-person. The provider focus group interviews are designed to collect information regarding providers' deprescribing practices. Specifically, 1) their attitudes about prescribing opioids and/or BZDs for their patients; (2) what recommendations they have for reducing the dosage of opioids and/or BZDs; (3) what recommendations they have for adjunctive therapies that can help support reducing the dosage; (4) what concerns they have in terms of dependence, withdrawal and/or overdose for their patients; and (5) what barriers, if any, they have experienced when recommending alternative therapies. The interviews will be guided by a standardized interview protocol. All interviews will be audio-taped to facilitate planned content analyses. We anticipate that the focus group interviews will take approximately 2 hours to complete. Resulting transcripts will be analyzed similarly to the patient focus group interviews.

3.1.2 Web-Based Survey:

We will collect primary data via a web-based survey of all health care providers within the intervention and control clinics. The web-based survey will be distributed at two different time points: 1) after clinics have been randomized into intervention and control groups but before the intervention is implemented; and 2) after the trial has been completed. The purpose of the survey is to collect information on the intervention's effectiveness.

We estimate that each clinic will employ an average of six health care providers. Thus, we estimate that we will request approximately 120 health care providers (6 providers per clinic x 20 clinics) to complete the survey. The survey will solicit information that is easily quantified (e.g., basic demographic data, terminal professional degree, clinic volume, perceived barriers to deprescribing services, and provider knowledge, skills and attitudes about deprescribing). We anticipate that the web-based survey will take an average of 15 minutes to complete.

Providers will not receive the survey until after randomization has occurred. A clinic's eligibility for randomization into the trial *or* continued participation in the trial is completely independent of whether or not any clinic providers respond to the survey.

3.1.3 Cluster-Randomized Trial:

This **cluster-randomized trial** will use a longitudinal pre-post research design. Data will be collected from two groups (1 intervention and 1 control) with three comparisons. The groups will be comprised of primary care outpatient clinics within the UNC HealthCare System, including family medicine, primary care, and geriatric clinics. Clinics will be randomly selected into either intervention or control clinics. Study team members will work with providers at intervention clinics to implement the educational intervention, which is designed to reduce falls risk through opioid and BZD deprescribing. Potentially eligible patients will be shared with physicians at intervention clinics. Emphasis will be placed on this list as a potential list; in no way are the study team mandating providers to de-prescribe opioids or BZDs in these patients. The decision to de-prescribe is left solely to the provider.

3.2 Study Duration, Enrollment and Number of Subjects

This study will last for 4 total years. Year 1 will consist of organizing the study team and conducting focus groups. In Year 2, clinics will be randomized, and the educational intervention will be finalized for testing. The cluster-randomized intervention will begin in the 7th month of year 2 and last through the 6th month of year 3. Data analysis will be conducted in the remainder of year 3 and into year 4.

We expect the following number of participants:

- Focus Groups: 12 patients, 12 providers
- Web-based surveys: 120 providers
- Cluster-randomized trial:
    - Number of clinics (intervention & control): 20;
    - Number of patients (intervention and control): 3,000

3.3 Study Population

Inclusion Criteria: To be eligible to participate in the study, clinics need to be affiliated with UNC HealthCare and have a patient population that includes patients 65 years of age or older. Targeted patients will be ambulatory (not institutionalized), aged at least 65 years old and taking at least one opioid or benzodiazepine medication listed in the design section for > 6 weeks, indicating they are at high risk for falls.

The UNC HealthCare outpatient clinics serve a diverse patient population, including women and minorities. The patient population served varies across clinics and geographical areas. UNC HealthCare coordinates care and manages care for more than 130,000 older adult patients in North Carolina. These beneficiaries are from all over the state. Females comprise 65.5% of the population. Approximately 52% are White, Non-Hispanic, 5.0% are Hispanic, 3.0% are Native American, 2.0% are Asian, and 38% are African American, Non-Hispanic.

To be eligible to participate in the patient focus group, participants need to be a patient in one of the intervention group clinics, 65 years of age and older, and taking at least one opioid or benzodiazepine chronically.

Exclusion Criteria:
Clinics that do not provide primary care services will be excluded from participating in the study.

Patients will be excluded from the focus groups if they are not English speaking, exhibit signs of cognitive impairment or speech/hearing deficits, are undergoing active cancer treatment, receiving hospice care, live in a skilled nursing facility, or do not wish to be audio-recorded.

Patients will be excluded from the cluster-randomized trial analysis if their EHR problem list indicates they have cancer, cognitive impairment, are receiving hospice care, live in a skilled nursing facility, or are non-English speaking.

4 STUDY PROCEDURES (what will be done)

4.1 Screening/Baseline Visit Procedures and Observation Procedures for the Focus Groups:

(1) **Patient Focus Groups: We** will conduct one focus group with 12 patients who are active users of opioids and/or BZDs prior to the implementation of the intervention. Patients will be recruited from intervention clinics. The focus group will be recorded and transcribed, data will be analyzed as described in Section 3.1.1. Results from the focus group will inform the design of the toolkit's patient education resources to support the deprescribing intervention.

(2) **Provider Focus Groups: We** will conduct one focus group with 12 healthcare providers who are actively engaged in treatment of patients who are taking opioids and BZDs. Providers will also be recruited from intervention clinics. The focus group will be recorded and transcribed, and data will be analyzed as described in Section 3.1.1. Results from the focus group will inform the design of the toolkit and associated resources to support the deprescribing intervention.

4.2 Screening/Baseline Visit Procedures and Observation Procedures for the Cluster-Randomized Trial:

(1) Randomize intervention and control clinics: A list of clinics that are interested in participating will be compiled by the primary investigators. A member of the study team, Ben Urick, PharmD, PhD, will use the random number generator built into SAS to randomize 10 clinics into treatment and 10 clinics into control groups. If the number of potentially eligible clinics exceeds 20, 20 clinics will be chosen at random, and this list will subsequently be divided into 10 random treatment clinics and 10 random controls. Results of randomization will be provided to the study coordinators, who will inform clinics of their status.

Data for all patients who meet inclusion criteria will be included in this analysis. This educational

intervention is intended to better inform providers in intervention clinics as to the fall-related risks associated with opioids and BZDs as well as best practices in deprescribing opioids and BZDs. A toolkit to support screening and deprescribing will also be provided to providers at intervention clinics. It is expected that providers will use this information to attempt deprescribing for any patients for whom they feel the benefits of deprescribing outweigh the risks. The study team will collect data from the EHR on all potentially eligible patients, regardless of any attempted deprescribing. The same data will be collected for patients who received care from treatment and control clinics.

(2) Screen for risk of falling: Intervention group clinics will be expected to screen all patients who appear on the baseline report for falls risk, questions if the patient has not already been screened previously, by using the STEADI screening questions. Each clinic will be allowed to determine how they will integrate these questions into the patient care process. Although the processes used to integrate the screening questions into patient care will vary across clinics, all clinics will be required to enter patient responses to the questions into the EHR, as is standard practice in all UNC HealthCare clinics.

(3) Educate about opioid and BZD medications as they relate to falls: Intervention Group clinics will be encouraged to educate all patients who appear on the baseline report for opioid and BZD use that may place them at increased risk for falls. The baseline report will include all patients in that clinic who are 65 years and older and on an opioid and/or BZD for > 6 weeks. Printed and online educational materials highlighting the risks associated with opioid and BZD use will be provided to clinics for their patients. Providers will be encouraged to use these materials in conjunction with verbal education.

(4) Educate on overall fall prevention strategies: Printed and online patient education materials regarding falls risk and falls prevention will be given to the intervention clinics to give to patients whom they believe would benefit from the materials. General patient education materials, not specific to opioids or benzodiazepines, will be given to all control clinics.

(5) Implement the Deprescribing Educational Intervention: A medication review is part of usual care conducted during a typical clinic visit. If utilized, the toolkit and associated resources can augment this process. During the medication review, the HCP (e.g. nurse, physician, pharmacist, etc.) will follow a consistent approach to patient care, reviewing the patient's medications and identifying medication therapy problems. Special attention will be given to the opioid and BZD medications that the patient is taking since these medications are the focus of this project. To improve the process of the deprescribing for opioids and BZDs, a guidance algorithm and deprescribing toolkit will be available for use. The deprescribing toolkit is being developed by the research team and is based upon evidence-based literature and clinical guidelines. For this study, we will use the toolkit to offer suggestions of medications that may assist with pain and sleep as alternatives to opioids and BZDs. We will also provide patient education materials for the clinics to give their patients. We will make revisions to the toolkit based on feedback from our provider focus group and will amend or supplement our patient education resources based on feedback from our patient focus group.

HCPs performing the opioid and BZD medication reviews will be trained by research personnel (*see Training of Personnel*) to use the toolkit to guide the assessment of opioid and BZD use, the assessment of the patient's risk of falls, and the development of recommendations including possible non-pharmacological interventions they can introduce. In all cases, however, the HCPs will be trained to use their clinical judgment and weigh the risk versus benefit of a medication change before making a recommendation. If the need for a reduction in opioid or BZD dose is warranted, the reasoning and potential solutions will be discussed with the patient. As part of this discussion, the HCP will assess patient willingness to make recommended changes to their medication regimen. As appropriate, patient preferences will be incorporated into recommendations implemented. Patients will receive follow-up from the clinic within a timeframe that fits with the deprescribing strategy employed and which the provider and patient agree upon. During the assessment phase, the provider will also solicit information from the patient concerning medical problems associated with an increased risk of falls (e.g., orthostatic hypotension, nocturia) and provide fall prevention education based on the STEADI resources provided in the toolkit.

(6) Training of Personnel. All personnel at Intervention Group clinics will be required to take part in an educational training session, which will include information about the study's purpose, general processes, and available resources. The training session will include physicians, physician assistants, nurse practitioners, nurses, pharmacists, technicians, medical assistants, office managers, case managers and quality improvement officers. Following the training, clinic staff will be able to (1) administer the three STEADI screening questions to all older adults identified in the baseline reports generated by Epic EHR data; (2) identify patients who qualify for the deprescribing intervention; (3) provide patients education about the risks associated with opioid use, BZD use, and falls; and (4) utilize the toolkit and associated resources to effectively and safely deprescribe opioids and BZDs when warranted. Special emphasis will be placed on sharing non-pharmacological recommendations with patients as alternatives to medications associated with an increased risk of falling. Finally, we also will provide in the toolkit - for clinicians interested in learning more - primary literature and other resources related to deprescribing.

(7) Following launch of the study, continuous quality improvement processes will be initiated. Specifically, a postdoctoral fellow based in the central project office will contact each intervention clinic within one week of the implementation of the intervention, then monthly thereafter. The phone contact will enable clinic personnel to: (1) ask and have answered any questions they may have about study procedures and (2) report unforeseen issues that need to be addressed, including safety concerns related to deprescribing. The fellow will also conduct clinic site visits if needed to resolve unforeseen problems and maintain engagement and fidelity for the duration of the project.

4.3 Subject Completion/ Withdrawal procedures

Any clinic wishing to withdraw from the intervention group can do so at any time.

4.4 Screen failure procedures

Providers in the intervention clinics will continue to provide usual care to all patients that do not screen into the study.

5 SCREENING AND MONITORING EVALUATIONS AND MEASUREMENTS (how measurements will be made)

In addition to the focus group instruments and web-based surveys, the development of which are detailed in Section 3, the following outcomes will be used for the cluster-randomized trial.

The primary outcome for this study is opioid and BZD exposure, as measured by dose and discontinuation rate. A secondary outcome variable is the rate of falls. We will also evaluate providers' satisfaction with the deprescribing toolkit. For clinicians providing services at an Intervention Group clinic, we will assess three outcome variables that reflect the process of service delivery: (1) whether or not their patients received education on opioids and BZD and their relationship to falls; (2) whether or not the deprescribing toolkit was utilized as part of the deprescribing process; and (3) whether or not HCPs referred to deprescribing toolkit recommendations concerning medication and non-medications to reduce the patient's risk of falls.

- **Opioid and BZD Exposure:** The primary outcome of this study will be exposure to opioid and/or BZD. This will be measured in two ways: (1) dose and (2) discontinuation. We will convert all opioid prescriptions to morphine milligram equivalents (MME), and all BZD prescriptions to diazepam milligram equivalents (DME). The method for calculating MME is well-established, and recommended by the CDC for evaluating opioid-related complications.[43] Calculating DME is analogous, and has been used in previous work to evaluate exposure to BZD and has been used previously to evaluate success of discontinuation interventions.[39,40] For each class of medications, we will calculate milligram equivalents for all observable prescriptions in the 12 months prior to the index and in the 12 months

post-index. To create a more conservative estimate of the impact of the intervention, we will assume that any authorized refills were filled. [DATA SOURCE: Epic EHR]

Discontinuation will be defined as a lack of opioid or BZD prescription from 181 to 360 days after the index. It is acknowledged that the intervention may not have an immediate impact on opioid and BZD discontinuation as prescribers taper patients' doses down, therefore a gap is given to allow for tapering in advance of discontinuation. [DATA SOURCE: Epic EHR]

- **Risk of Falls:** The secondary outcome of this study will be patients' falls risk, as identified by patients' recorded responses to the STEADI questions embedded in the EHR and by the rate of falls during the one-year post-intervention period compared to the one-year pre-period. We will obtain information regarding falls using data from Epic EHR, and research team members will record the number of injurious falls-related visits made during pre-intervention and post-intervention periods. [DATA SOURCE: Epic EHR]

6 STATISTICAL CONSIDERATION

Statistical considerations are described below for the primary and secondary outcomes from the cluster-randomized trials. No statistical analysis will be conducted with focus groups, and the surveys will be analyzed using descriptive statistics and bivariate assessments for pre-post change in provider knowledge, skills, and attitudes in management of opioids and BZDs between intervention and control clinics.

All hypothesis tests that are observed and deemed to be not statistically significant will be reported as being inconclusive. All statistical estimates of population parameters will be tabulated along with corresponding confidence intervals (CIs) and/or standard errors (SE). All hypothesis tests and tests for power are two-sided with an overall alpha of 0.05, unless otherwise specified.

6.1 Primary Outcomes: Opioid and BZD exposure, as defined by changes in milligram equivalents over time across control and intervention groups as well as discontinuation of opioids or BZDs. Outcomes are described in more detail in Section 6.3.

6.2 Secondary Outcomes: Risk of Falls, as defined by differences in rate of falls over time across control and intervention groups and described in more detail in Section 6.3.

6.3 Statistical Methods

Primary Analyses: We will examine between-group differences in opioid and BZD exposure associated with an increased risk of falls in the intervention and control groups. Analyses will be performed using the following intention-to-treat principle, and index dates for the intervention group will correspond to the date on which they first received any intervention-related clinical activity. Index dates for otherwise eligible patients from the control group will be chosen at random from each patients' set of clinic visits corresponding to an eligible intervention visit during the study period. Our primary hypotheses are:

- Relative to individuals in both control groups, individuals in both intervention groups will experience a significant reduction in the milligram equivalent exposure to opioids and BZDs during the 1-year follow-up period.

- Relative to individuals in both control groups, individuals in both intervention groups will experience a significantly greater discontinuation rate of opioids and BZDs during the follow-up period 181 to 360 days after index.

There are two overlapping groups of patients aged 65+ that will be included in this study: opioid users and BZD users. For each hypothesis, there will be separate models comparing the impact of the program on opioid users and BZD users. Patients who use both will appear in both models. To examine any differential impact of the program among patients who use both classes of medications, a sensitivity analysis will be performed and will include an indicator variable for patients who use both classes.

Generalized estimating equations (GEE, PROC GENMOD) will be used to create longitudinal pre-post model for the milligram equivalent exposure models. An identify link with a normal distribution will be used for milligram equivalent exposure. The functional form of the model is as follows:

$$\text{Eq. 1: } Y = \beta_0 + \beta_1 X_{exp} + \beta_2 X_{period} + \beta_3 X_{exp*period} + \beta_n X_n + \epsilon$$

Where X_{exp} is the exposure group indicator, X_{period} is the pre-post indicator, and $X_{exp*period}$ is the period-by-treatment interaction indicator and, correspondingly, β_3 is the parameter of the difference in outcome by period for the intervention group compared to the control. X_n represents a vector of covariates.

Secondary Analyses: Our secondary **hypothesis is:**

- Relative to individuals in both control groups, individuals in both intervention groups will experience a reduction in the risk of experiencing a fall following the index date.

Risk is operationalized as the odds of experiencing a fall in the 12-month post-index compared to the 12 months pre-index. The models used for this analysis match those in Equation 1, with the exception that the models for fall risk will use a log link with a binomial distribution instead of an identity link with a normal distribution.

6.4 Sample Size and Power:

6.4.1 Milligram equivalent exposure to opioids

Effect of various interventions on milligram equivalent exposure to opioids has shown mean reductions ranging from 31% to 49%.[44,45] A GEE model (continuous outcome) with a total sample size of 3000 patients, each measured two times before and after intervention will have statistical power in the range of 0.865-0.999 to detect differences in means of 0.30 or higher in milligram equivalent exposure when the standard deviation is in the range of 1 to 3. Pre-period measurements will include the 12-month period prior to index, and post-period measurements will include the 12 months following index. This assumes that the baseline correlation between observations is 0.2 or higher and a 20% attrition rate will be observed. Another assumption is that we will assign subjects randomly to an intervention group and control group with an equal split between the two groups. Notably, if the standard deviation of observations is above 3.0 or if the attrition rate is greater than 20%, then our statistical power will drop significantly. All calculations were performed using PASS Software.[46]

6.4.2 Discontinuation rate of opioids

According to a recent systematic review of literature[47], most opioid continuation rates are from relatively unreliable studies and can be anywhere between 54% to 100%. However, a recent study[48] has found the odds ratio of opioid discontinuation in control versus treatment group to be 1.5 (95% CI [1.0-2.1]). If clinics are randomly assigned to a treatment group and a control group, with an equal number of patients in both groups, a GEE model (binary outcome) with a total sample size of 3000

patients, each measured two times before and after intervention, will have statistical power in the range of 0.631-0.953 to detect differences in odds ratios in the range of 1.30-2.0. There is significant drop in power for detecting differences in odds ratios below 1.3. Baseline probabilities in the treatment and control group are assumed to be in the range of 0.020-0.050 and we assumed a 20% attrition rate. For lower baseline probabilities of 0.010 in each group, the statistical power is in the range of 0.485-0.860 for detecting odds ratios of 1.3 or higher.

6.4.3 Milligram equivalent exposure to BZDs

Effect of various interventions on milligram diazepam equivalent per day exposure to BZDs has shown mean reductions ranging from 25% to 85%.[49,50] If clinics are randomly assigned to a treatment group and a control group, with an equal number of patients in both groups, a GEE model with a total sample size of 3000 patients, each measured two times before and after intervention, will have statistical power in the range of 0.678-0.969 to detect differences in means of 0.25 or higher in milligram equivalent exposure when the standard deviation is below 2. Pre-period measurements will include the 12-month period prior to index, and post-period measurements will include the 12 months following index. This assumes that the baseline correlation between observations is 0.2 or higher and that we will observe a 20% attrition rate. Power increases for larger mean difference values and drops significantly at higher standard deviation values.

6.4.4 Discontinuation rate of BZDs

A systematic review of literature has demonstrated discontinuation rates between 27-80% for interventions to reduce exposure to benzodiazepines.[51] If clinics are randomly assigned to a treatment group and a control group, with an equal split between the two groups, a GEE model with a total sample size of 3000 patients, each measured two times before and after intervention, will have statistical power in the range of 0.859-1.000 to detect differences in post-intervention event probabilities in the range of 0.25-0.85. There is significant drop in power for detecting group differences below 0.25. Baseline probabilities in the treatment and control group are assumed to be in the range of 0.010-0.050 and we assumed a 20% attrition rate.

6.5 Interim Analysis

No interim analyses of the study are planned.

6.6 Control of Bias

Both statistical controls and design elements are used to control for bias in the measurement of study outcomes. The longitudinal pre-treatment post-treatment design helps to control for natural rate of deprescribing in a prevalent user population.

6.7 Handling of Missing Values

We will apply no imputation method to this data. All data are observational, and randomization should balance missingness across samples. As such, it is unlikely that missing values will create selection bias. Additionally, our chosen statistical method allows for estimation of longitudinal effects even if patients have a missing outcome value.

7 SAFETY MANAGEMENT

The primary mechanism for safety monitoring will be monthly check-ins by a postdoctoral research fellow who is part of the study team. As a part of monthly check-in calls, the fellow will inquire about any reported adverse effects from opioid and BZD deprescribing, such as increased pain, anxiety and

trouble sleeping. The educational intervention will include instructions for providers to notify clinic staff of any adverse effects, such as withdrawal. Additionally, the instructions will state that the provider should notify the study team directly if any serious adverse events, such as seizures from BZD, occur.

Withdrawal symptoms are a normal and common result of deprescribing. Prescribers of these medicines are familiar with these symptoms, and additional information on withdrawal will also be included in the educational materials. Options for patients who experience adverse effects from deprescribing include contacting the nighttime call service, walk-in clinics, visiting the emergency department, and same-day service at providers' offices. These usual sources of care will be available to assist patients if the need arises.

8 DATA COLLECTION AND MANAGEMENT

Data collection and management of focus group and web-based survey data are described in Section 3. Additionally, data will be held in a password-protected folder on a shared drive accessible to all members of the study team involved with analysis and interpretation of focus group results.

For the cluster-randomized trial, data for the primary and secondary analyses will be gathered from the EHR at baseline and after the 1-year intervention. The primary and secondary outcomes are observational and do not require direct data collection from patients and providers. All information needed for these analyses will be recorded as a part of usual care. Data will be stored on a secure, HIPAA-compliant research workspace managed by Research Computing, a division of UNC Information Technology Services (ITS). The server requires an ONYEN and a two-factor authentication to log on, which is managed by ITS. There are two level of users, Level 1 and Level 2. Level 1 users will have access to all identifiable data, and Level 2 will have access only to coded datasets.

Identifiable data will be collated by the Carolina Data Warehouse for Health (CDWH) and loaded onto the server by a member of the study team, Ben Urick, PharmD, PhD. All data from CDWH will be received as comma-separated values (CSV) files and will be converted into SAS databases when loaded onto the secure server. He will replace any personal identifiers (e.g. medical record number, beneficiary ID number, name, etc.) with a randomly generated patient ID specific to the study. He will load data into a folder accessible to lower-level users who cannot access or view the fully identified files. All users will be limited to members of the study team and will include only those members who need to analyze or interpret data. Ben Urick, PharmD, PhD will lead the analysis, with support by Neepa Ray, MS and Shweta Pathak, MPH, PhD.

Dr. Urick will oversee data quality efforts. Data from surveys will be captured and stored to support easy retrieval. Codebooks containing variable names and descriptions will be created for all files. The codebooks will be stored on the same server as the data. Missing responses in surveys will be identified, and consideration will be given as to discarding a partially complete response vs. including items to which a participant responded when data are analyzed. Neepa Ray has nearly a decade of experience managing datasets for clinical and observational studies, will lead efforts to clean the EHR data and prepare data for analysis. A record of linkages between EHR datasets will be maintained and kept in the same folder as the codebook. Missing data will be explored, and any missingness that appears unbalanced between the intervention and control clinics will be reported to the study team for consideration. When data are analyzed, Dr. Urick will ensure that all coders adhere to the pre-established codebook and expand upon the codebook when new derived variables are added to the database.

Data for the outcomes in Aim 3 will be collected from providers via the web-based survey.

9 RECRUITMENT STRATEGY

Clinics will be identified from patient data pulled from UNC HealthCare's EHR (Epic). Clinic recruitment will be facilitated by Dr. Jan Busby-Whitehead. Dr. Busby-Whitehead, with other team members from the UNC Medical School, will reach out to key stakeholders in the targeted clinics (practice managers &/or physicians). They will provide each clinic with a letter of invitation to participate in the study and will meet with the key stakeholders in person to discuss any questions they have about the nature of the study, procedures, risks, and benefits. A copy of the study's IRB application will also be provided to these stakeholders if requested.

No patient recruitment for participation in the study (except for focus group participants) will be warranted since the study's intervention will be focused on educating providers on improving their delivery of the standard of care.

Provider focus group participants will be identified by the research team and recruited from intervention clinics. They will be sent letters of recruitment and will be contacted in person by Dr. Jan Busby-Whitehead or other members of the research team.

Patient focus group participants will be identified by Epic data and recruited from intervention clinics. They will be sent letters of recruitment from the research team.

10 CONSENT PROCESS

Clinics: Dr. Jan Busby-Whitehead, with other team members from the UNC Medical School, will reach out to key stakeholders in the targeted clinics (practice managers and/or physicians). They will provide a letter of invitation to participate in the study and will meet with these key stakeholders in person to discuss any questions the stakeholders have about the nature of the study, procedures, risks, and benefits. A copy of the study's IRB application will be provided to these stakeholders if requested. Each clinic's 'study champion' will be asked to sign a consent form prior to the clinic's participation. Consent from clinic patients will not need to be obtained since the study's intervention will be focused on educating providers on improving their delivery of the standard of care.

Providers in participating clinics, who will be asked to complete two web-based surveys, will be provided a written consent form that they will need to submit electronically prior to being able to access the survey questions. This consent form will include a written statement stating that their participation in the survey is voluntary and by completing the survey they are consenting to participate. The consent form will also delineate the nature of the study and the risks and benefits of participating.

Focus group participants will be provided a written consent form that they will need to sign prior to the start of the focus group session. The consent forms will delineate the nature of the study and the risks and benefits of participating in the focus group.

11 PLANS FOR PUBLICATION

The UNC study team will work with the CDC through the cooperative agreement to determine an appropriate dissemination plan. At this time, we will present at national pharmacy and interprofessional geriatric meetings. We also plan to present the research in peer-reviewed journals.

12 REFERENCES

1. Falls Prevention Facts. National Council on Aging. Available at: https://www.ncoa.org/news/resources-for-reporters/get-the-facts/falls-prevention-facts/ Accessed March 22, 2018.
2. Florence CS, Bergen G, Atherly A, et al. Medical Costs of Fatal and Nonfatal Falls in Older Adults. J Am Geriatr Soc. 2018; Available ahead of print: https://doi.org/10.1111/jgs.15304
3. Gillespie LD, Robertson MC, Gillespie WJ, et al. Interventions for preventing falls in older people living in the community. Cochrane Database Syst Rev. 2012;9:CD007146.
4. Jones TS, Ghosh TS, Horn K, Smith J, Vogt RL. Primary care physicians perceptions and practices regarding fall prevention in adult's 65 years and over. Accid Anal Prev. 2011;43(5):1605-1609.
5. Blalock SJ, Casteel C, Roth MT, Ferreri S, Demby KB, Shankar V. Impact of enhanced pharmacologic care on the prevention of falls: a randomized, controlled trial. Am J Geriatr Pharmacother 2010;8(5):428-440.
6. Casteel C, Blalock SJ, Ferreri S, Roth MT, Demby KB. Implementation of a community pharmacy-based falls prevention program. Am J Geriatr Pharmacother, 2011;9(5):310-9. PMID: 21925959
7. Ferreri S, Roth MT, Casteel C, Demby KB, Blalock SJ. Methodology of an ongoing, randomized controlled trial to prevent falls through enhanced pharmaceutical care. Am J Geriatr Pharmacother. 2008;6(2):61-81.
8. Self-reported falls and fall-related injuries among persons aged at least 65 years – United States, 2006. *Morbidity and Mortality Weekly Report* 2008;57:225-229.
9. Alexander BH, Rivara FP, Wolf ME. The cost and frequency of hospitalization for fall-related injuries in older adults. *Am J Public Health.* 1992;82(7):1020-1023.
10. Finlayson ML, Peterson EW. Falls, aging, and disability. *Phys Med Rehabil Clin N Am.* 2010;21(2):357-373.
11. Gill TM, Murphy TE, Gahbauer EA, Allore HG. The course of disability before and after a serious fall injury. *JAMA Intern Med.* 2013;173(19):1780-1786.
12. Gill TM, Murphy TE, Gahbauer EA, Allore HG. Association of injurious falls with disability outcomes and nursing home admissions in community-living older persons. *Am J Epidemiol.* 2013;178(3):418-425.
13. Hartholt KA, van Beeck EF, Polinder S, et al. Societal consequences of falls in the older population: injuries, healthcare costs, and long-term reduced quality of life. *J Trauma.* 2011;71(3):748-753.
14. Stevens JA, Corso PS, Finkelstein EA, Miller TR. The costs of fatal and non-fatal falls among older adults. *Inj Prev.* 2006;12(5):290-295.
15. Leipzig RM, Cumming RG, Tinetti ME. Drugs and falls in older people: a systematic review and metaanalysis: II. Cardiac and analgesic drugs. *J Am Geriatr Soc.* 1999;47(1):40-50.
16. Leipzig RM, Cumming RG, Tinetti ME. Drugs and falls in older people: a systematic review and metaanalysis:I. Psychotropic drugs. *J Am Geriatr Soc.* 1999;47(1):30-39.
17. Cumming RG. Epidemiology of medication-related falls and fractures in the elderly. *Drugs Aging.*1998;12(1):43-53.
18. Ensrud KE, Blackwell TL, Mangione CM, et al. Central nervous system-active medications and risk for falls in older women. J Am Geriatr Soc. 2002;50(10):1629-1637.
19. Mustard CA, Mayer T. Case-control study of exposure to medication and the risk of injurious falls requiring hospitalization among nursing home residents. *Am J Epidemiol*. 1997;145(8):738-745.
20. Ray WA. Psychotropic drugs and injuries among the elderly: a review. *J Clin Psychopharmacol.* 1992;12(6):386-396.
21. Huang AR, Mallet L, Eguale T, et al. Medication-related falls in the elderly: causative factors and preventive strategies. *Drugs Aging* 2012;29(5):359-76.
22. Aguiluz J, Alvares M, Pimentel, et al. How to face a patient with benzodiazepine dependence in primary health care? Strategies for withdrawal. *Medwave* 2018;18(1):e7159.
23. Markota M, Rummans TA, Bostwick JM, et al. Benzodiazepine use in older adults: dangers, management, and alternative therapies. *Mayo Clin Proc* 2016;91(11):1632-39.
24. Mott DA, Martin B, Breslow R, et al. Impact of a medication therapy management intervention targeting medications associated with falling: Results of a pilot study. *J Am Pharm Assoc (2003).* 2016;56(1):22-28.

25. Weiner DK, Hanlon JT, Studenski SA. Effects of central nervous system polypharmacy on falls liability in community-dwelling elderly. *Gerontology* 1998;44(4):217-21.
26. Milos V, Bondesson Å, Magnusson M, Jakobsson U, Westerlund T, Midlöv P. Fall risk increasing drugs and falls: a cross-sectional study among elderly patients in primary care. *BMC Geriatr* 2014 Mar 27;14:40. doi:10.1186/1471-2318-14-40.
27. Farrell B, Tsang C, Raman-Wilms L, Irving H, Conklin J, Pottie K. What are priorities for deprescribing for elderly patients? Capturing the voice of practitioners: a modified delphi process. *PLoS One* 2015 Apr 7;10(4):e0122246. doi: 10.1371/journal.pone.0122246. eCollection 2015.
28. Steinman MA, Beizer JL, DuBeau CE, Laird RD, Lundebjerg NE, Mulhausen P. How to use the AGS 2015 Beers Criteria – A guide for patients, clinicians, health systems, and payors. *J Am Geriatr Soc 2015 Dec;63(12): e1–e7.* doi:10.1111/jgs.13701.
29. Kahan M, Wilson L, Mailis-Gagnon A, Srivastava A, National Opioid Use Guideline Group. Canadian guideline for safe and effective use of opioids for chronic noncancer pain: clinical summary for family physicians. Part 2: special populations. *Can Fam Physician* 2011 Nov;57(11):1269-76, e419-28.
30. Kimura T, Ogura F, Yamamoto K, Uda A, Nishioka T, Kume M, Makimoto H, Yano I, Hirai M. Potentially inappropriate medications in elderly Japanese patients: effects of pharmacists' assessment and intervention based on Screening Tool of Older Persons' Potentially Inappropriate Prescriptions criteria ver.2. J *Clin Pharm Ther* 2017 Apr;42(2):209-214. doi:10.1111/jcpt.12496.
31. Salonoja M, Salminen M, Aarnio P, Vahlberg T, Kivelä SL. One-time counselling decreases the use of benzodiazepines and related drugs among community-dwelling older persons. *Age Ageing* 2010 May;39(3):313-9. doi:10.1093/ageing/afp255.
32. Browne C, Kingston C, Keane C. Falls prevention focused medication review by a pharmacist in an acute hospital: implications for future practice. *Int J Clin Pharm* 2014 Oct;36(5):969-75. doi:10.1007/s11096-014-9980-3. Epub 2014 Aug 10.
33. Hill KD, Wee R. Psychotropic drug-induced falls in older people: a review of interventions aimed at reducing the problem. *Drugs Aging* 2012 Jan 1;29(1):15-30. doi:10.2165/11598420-000000000-00000.
34. Gregg JA, Tyson RL, Cook D. Benzodiazepines and geriatrics: What clinical practice strategies can be used to reduce the inappropriate prescribing? *Rehabil Nurs* 2016 Sep;41(5):270-5. doi:10.1002/rnj.288. Epub 2016 Jun 25.
35. Summary of the updated American geriatrics society/British geriatrics society clinical practice guideline for prevention of falls in older persons. *Journal of the American Geriatrics Society.* 2010;59:148-157.
36. Wenger NS, Solomon DH, Roth CP, et al. The quality of medical care provided to vulnerable community dwelling older patients. *Ann Intern Med.* 2003;139(9):740-747.
37. Stevens JA, Ballesteros MF, Mack KA, Rudd RA, DeCaro E, Adler G. Gender differences in seeking care for falls in the aged Medicare population. *Am J Prev Med.* 2012;43(1):59-62.
38. Eckstrom E, Neal MB, Cotrell V, Casey CM, McKenzie G, Morgove MW, DeLander GE, Simonson W, Lasater K. An interprofessional approach to reducing the risk of falls through enhanced collaborative practice. *J Am Geriatr Soc* 2016 Aug;64(8):1701-7. doi:10.1111/jgs.14178. Epub 2016 Jul 28.
39. Tannenbaum C, Martin P, Tamblyn R, et al. Reduction of Inappropriate benzodiazepoine prescriptions among older adults through direct patient education: The EMPOWER cluster randomized trial. *JAMA Intern Med*. 2014;174(6):890-898. doi:10.1001/jamainternmed.2014.949
40. Paquin AM, Zimmerman K, Rudolph JL. *Expert Opin. Drug Sa*f. (2014) 13(7):919-934.
41. Guaiana G and Barbui C. Discontinuing benzodiazepines: best practices. *Epidemiology and Psychiatric Sciences* (2016), 25, 214–216. 2016 doi:10.1017/S2045796016000032
42. Centers for Disease Control and Prevention. STEADI – Older Adult Fall Prevention. CDC Web Site. Available at: https://www.cdc.gov/steadi/. Accessed April 10, 2019.
43. Calculating Total daily dose of opioids for safer dosage. https://www.cdc.gov/drugoverdose/pdf/calculating_total_daily_dose-a.pdf Available from the Centers for Disease Control and Prevention www.cdc.gov/drugoverdose/prescribing/guideline.htm Accessed March 22, 2018.
44. Ackerman AL, O'Connor PG, Doyle DL, et al. Association of an Opioid Standard of Practice Intervention With Intravenous Opioid Exposure in Hospitalized Patients. JAMA Intern Med. 2018;178(6):759–763. doi:10.1001/jamainternmed.2018.1044

45. Meisenberg BR, Grover J, Campbell C, Korpon D. Assessment of Opioid Prescribing Practices Before and After Implementation of a Health System Intervention to Reduce Opioid Overprescribing. JAMA Netw Open. 2018;1(5):e182908. doi:10.1001/jamanetworkopen.2018.2908
46. PASS 16 Power Analysis and Sample Size Software (2018). NCSS, LLC. Kaysville, Utah, USA, ncss.com/software/pass.
47. Frank, J. W., Lovejoy, T. I., Becker, W. C., Morasco, B. J., Koenig, C. J., Hoffecker, L., … Krebs, E. E. (2017). Patient Outcomes in Dose Reduction or Discontinuation of Long-Term Opioid Therapy. Annals of Internal Medicine, 167(3), 181. https://doi.org/10.7326/m17-0598
48. Liebschutz, J. M., Xuan, Z., Shanahan, C. W., LaRochelle, M., Keosaian, J., Beers, D., … Lasser, K. E. (2017). Improving Adherence to Long-term Opioid Therapy Guidelines to Reduce Opioid Misuse in Primary Care. JAMA Internal Medicine, 177(9), 1265. https://doi.org/10.1001/jamainternmed.2017.2468
49. Cabelguenne, D., Picard, C., Lalande, L., Jonker, J., Sautereau, M., Meunier, F., & Zimmer, L. (2018). Benzodiazepine dose reduction in prisoner patients: 15 years' teamwork between psychiatrists and pharmacists. Journal of Clinical Pharmacy and Therapeutics, 43(6), 807–812. https://doi.org/10.1111/jcpt.12709
50. Allison M Paquin, Kristin Zimmerman & James L Rudolph (2014) Risk versus risk: a review of benzodiazepine reduction in older adults, Expert Opinion on Drug Safety, 13:7, 919-934, DOI: 10.1517/14740338.2014.925444
51. Reeve, E., Ong, M., Wu, A., Jansen, J., Petrovic, M., & Gnjidic, D. (2017). A systematic review of interventions to deprescribe benzodiazepines and other hypnotics among older people. European Journal of Clinical Pharmacology, 73(8), 927–935. https://doi.org/10.1007/s00228-017-2257-8
