# Supplementary material for: A Pharmacist Consultant Service for Deprescribing Opioids and Benzodiazepines in Older Adults: A Cluster Randomized Trial
Source: JAMA Netw Open. 2026 Feb 26;9(2):e2560581. doi: 10.1001/jamanetworkopen.2025.60581 (PMC12947021; doi:10.1001/jamanetworkopen.2025.60581)
Supplement: Supplement 2. — eTable 1. Clinic Characteristics eTable 2. List of Relevant Indications for Opioid and Benzodiazepine (BZD) Use eTable 3. Prescribing Data by Generic Drug Name Among Older Adults (65+) From Enrolled Primary Care Practices eTable 4. Average Daily Morphine Milligram Equivalents by Clinic (Pre- and Post-intervention) eTable 5. Intervention Effectiveness for Secondary Outcomes eTable 6. Average Daily Diazepam Milligram Equivalents by Clinic (Pre- and Post-intervention) eTable 7. Effectiveness of Opioid Deprescribing Intervention on Average Daily Morphine Milligram Equivalents - High vs. Low Baseline Exposure eTable 8. Effectiveness Benzodiazepine Deprescribing Intervention on Average Daily Diazepam Milligram Equivalents – High vs. Low Baseline Exposure eTable 9. Effectiveness of Opioid Deprescribing Intervention on Average Daily Morphine Milligram Equivalents – Older or Younger Than 80 Years of Age [file jamanetwopen-e2560581-s002.pdf]

Supplemental Online Content

Busby-Whitehead J, Ferreri SP, Niznik J, et al. A pharmacist consultant service for deprescribing opioids and benzodiazepines in older adults: a cluster randomized trial. *JAMA Netw Open*. 2026;9(2):e2560581. doi:10.1001/jamanetworkopen.2025.60581

eTable 1. Clinic Characteristics
eTable 2. List of Relevant Indications for Opioid and Benzodiazepine (BZD) Use
eTable 3. Prescribing Data by Generic Drug Name Among Older Adults (65+) From Enrolled Primary Care Practices
eTable 4. Average Daily Morphine Milligram Equivalents by Clinic (Pre- and Post-intervention)
eTable 5. Intervention Effectiveness for Secondary Outcomes
eTable 6. Average Daily Diazepam Milligram Equivalents by Clinic (Pre- and Post-intervention)
eTable 7. Effectiveness of Opioid Deprescribing Intervention on Average Daily Morphine Milligram Equivalents - High vs. Low Baseline Exposure
eTable 8. Effectiveness Benzodiazepine Deprescribing Intervention on Average Daily Diazepam Milligram Equivalents – High vs. Low Baseline Exposure
eTable 9. Effectiveness of Opioid Deprescribing Intervention on Average Daily Morphine Milligram Equivalents – Older or Younger Than 80 Years of Age

This supplemental material has been provided by the authors to give readers additional information about their work.

© 2026 Busby-Whitehead J et al. *JAMA Network Open*. This is an open access article distributed under the terms of the CC-BY-NC-ND License, which does not permit alteration or commercial use, including those for text and data mining, AI training, and similar technologies.

eTable1. Clinic Characteristics

Clinic Name	65+ Population	Clinic Volume Opioids	Clinic Volume BZDs	Rural/Non-rural	Total providers	%MD Providers
Intervention 1	1583	75 (4.7%)	54 (3.4%)	Rural	8	62.5%
Intervention 2	2299	207 (9.0%)	155 (6.7%)	Rural	10	50%
Intervention 3	1214	56 (4.6%)	87 (7.2%)	Non-rural	4	0%
Intervention 4	1321	26 (2.0%)	31 (2.3%)	Rural	3	33.3%
Intervention 5	660	85 (12.9%)	54 (8.2%)	Rural	2	50%
Intervention 6	827	34 (4.1%)	46 (5.5%)	Rural	2	50%
Intervention 7	1544	7 (0.5%)	50 (3.2%)	Non-rural	6	33.3%
Intervention 8	803	84 (10.5%)	59 (7.3%)	Rural	3	33.3%
Control 1	3259	120 (3.7%)	102 (2.8%)	Rural	11	100%
Control 2	2634	64 (2.4%)	147 (5.6%)	Non-rural	11	81.8%
Control 3	3486	70 (2.0%)	84 (2.4%)	Non-rural	10	20%
Control 4	1390	46 (3.3%)	102 (7.3%)	Rural	4	80%
Control 5	1578	22 (1.4%)	29 (1.8%)	Rural	5	25%
Control 6	1360	8 (0.6%)	42 (3.1%)	Non-rural	5	40%
Control 7	1162	57 (4.9%)	65 (5.6%)	Rural	4	75%

© 2026 Busby-Whitehead J et al. *JAMA Network Open*. This is an open access article distributed under the terms of the CC-BY-NC-ND License, which does not permit alteration or commercial use, including those for text and data mining, AI training, and similar technologies.

eTable2. List of relevant indications for opioid and benzodiazepine (BZD) use.

Conditions	ICD-9	ICD-10	Relevance to Opioids vs. BZDs
**Major Depression**	296.2x, 296.3x	F32, F32.0, F32.1, F32.2, F32.3, F32.4, F32.5, F32.9, F33, F33.0, F33.1, F33.2, F33.3, F33.4, F33.40, F33.41, F33.42, F33.8, F33.9	BZD
**PTSD**	309.81	F43.1, F43.10, F43.11, F43.12	BZD
**Other Anxiety**	300.00, 300.01, 300.02, 300.09, 300.10, 300.20, 300.21, 300.22, 300.23, 300.29	F06.4, F40, F40.0, F40.00, F40.01, F40.02, F40.1, F40.10, F40.11, F40.2, F40.21, 40.22, F40.23, F40.24, F40.29, F40.8, F40.9, F41, F41.0, F41.3, F41.8, F41.9, F42, F43, F43.0, F43.8, F43.9, F45.2, F45.20, F45.21, F45.29, R45.7	BZD
**Alcohol-related disorder**	303.0x, 303.9x, 305.0x	F10.1X, F10.2X, F10.9x	BZD
**Opioid use disorder**	304.0x, 304.7x, 305.5x, excluding 304.03,304.73, 305.53	F11.1X, F11.2X	Opioid
**Insomnia**	307.4x, 327.00, 327.01, 327.02, 327.09, 780.51, 780.52	G47.00. G47.01. G47.09	BZD
**Chronic Pain**	256.60, 307.80, 307.81, 307.89, 346.0-346.9, 355.0, 355.9, 356.0, 357.2, 357.9, 710.0-739.9, 784.0	Musculoskeletal - A18.01-A18.02, A52.16, D48.1, E08.61x, E09.61x, E10.61x, E11.61x, E13.61x, M00-M02, M04.02-M04.09, M05-M19, M1A, M20.10, M21.61-M21.62, M22-M25, M32-M36, M43.2-M43.8X9, M45-M48, M49.80, M50, M51, M53, M54, M60.0-M60.2, M61-M63, M65-M67, M70-M72, M75-M77, M79, M96.1, M99.2-M99.7, N20.0, Q68.6, R25.2, R26.2, R29.8x Neuropathic - A52.15, B02 (exclude B02.1), EXX.4, EXX.610, EXX.65 (where X in “08”-“13”), E10.4, F45.42, G13.0, G13.1, G32.0, G35, G50- G52.1, G54-G59, G61.8, G61.9, G62.8, G62.9, G63-G65, G89.0, G90.0, G90.5, G95, G99.0-G99.2, M05.5, M54.13-M54.18, M54.3, M54.4, M60.8, M60.9, M79.1, M79.2, M79.7	Opioid
**Other Chronic Pain**	307.87, 337.0, 337.1, 338.0, 338.2, 338.4, 339.x, 354.4, 355.x-357.x, 377.x	G89, R52	Opioid

© 2026 Busby-Whitehead J et al. *JAMA Network Open*. This is an open access article distributed under the terms of the CC-BY-NC-ND License, which does not permit alteration or commercial use, including those for text and data mining, AI training, and similar technologies.

eTable3. Prescribing Data by Generic Drug Name Among Older Adults (65+) from Enrolled Primary Care Practices

**Opioids**	**n= 961* n(%)**	**Average daily exposure within drug+ (MMEs)**	**Total 1-year exposure in sample# (MMEs)**
tramadol	471 (49.0%)	8.2 (7.)	1409617.7
hydrocodone	363 (37.8%)	10.9 (12.5)	1444312.9
oxycodone	327 (34.0%)	28.9 (43.5)	3444539.5
codeine	50 (5.2%)	5.8 (8.7)	106377.1
morphine	23 (2.4%)	30.6 (32.3)	256860.0
fentanyl (transdermal)	18 (1.9%)	99.7 (97.1)	654866.4
methadone	10 (1.0%)	187.5 (131.5)	684450.0
hydromorphone	12 (1.3%)	36.2 (44.6)	158617.7
buprenorphine (transdermal)	5 (0.5%)	10.3 (10.9)	18837.0
oxymorphone	2 (0.2%)	133.3 (150.9)	97320.0
Receiving >1 medication	268 (27.9%)		
**Benzodiazepines**	**n= 1107* n(%)**	**Average daily exposure within drug+ (DMEs)**	**Total 1-year exposure in sample# (DMEs)**
alprazolam	427 (38.6%)	5.9 (6.7)	915217.4
clonazepam	277 (25.0%)	15.2 (13.2)	1531732.2
lorazepam	260 (23.5%)	4.3 (4.7)	412282.3
diazepam	112 (10.1%)	3.6 (3.84)	146978.4
temazepam	94 (8.5%)	1.7 (1.20)	58260.0
clorazepate	12 (1.1%)	4.4 (4.40)	19087.5
chlordiazepoxide	9 (0.8%)	2.8 (3.23)	9168.2
triazolam	5 (0.5%)	0.1 (0.06)	187.8
oxazepam	2 (0.2%)	4.4 (0.09)	3231.0
estazolam	1 (0.2%)	9.9 (N/A)	3620.0
Receiving >1 medication	87 (7.9%)		

*Frequencies represent the number of patients with a prescription order for a given medication.
+Among all prescription orders for that drug, the average daily MMEs or DMEs ordered.
#Among all prescription orders for that drug, the total number of MMEs or DMEs ordered.

© 2026 Busby-Whitehead J et al. *JAMA Network Open*. This is an open access article distributed under the terms of the CC-BY-NC-ND License, which does not permit alteration or commercial use, including those for text and data mining, AI training, and similar technologies.

eTable4. Average Daily Morphine Milligram Equivalents by Clinic (pre- and post-intervention)

Clinic Name	Receiving Any Opioids Pre-intervention n=961	Receiving Any Opioids Post-intervention n=915	Average daily MMEs Pre-Intervention	Average daily MMEs Post-Intervention	MMEs >50 Pre-Intervention	MMEs >50 Post-Intervention
Intervention 1	75 (7.8%)	72 (7.5%)	45.7	45.0	10 (13.3%)	12 (16.0%)
Intervention 2	207 (21.5%)	197 (20.5%)	30.2	27.1	40 (19.3%)	36 (17.4%)
Intervention 3	56 (5.8%)	51 (5.3%)	7.7	8.2	0	1 (1.8%)
Intervention 4	26 (2.7%)	25 (2.6%)	9.5	7.3	0	0
Intervention 5	85 (8.8%)	83 (8.6%)	29.81	25.5	12 (14.1%)	14 (16.5%)
Intervention 6	34 (3.5%)	31 (3.2%)	12.5	10.9	0	0
Intervention 7	7 (0.7%)	6 (0.6%)	5.9	6.9	0	0
Intervention 8	84 (8.7%)	81 (8.4%)	25.2	21.9	10 (11.9%)	8 (9.5%)
Control 1	120 (12.5%)	116 (12.1%)	23.4	21.9	14 (11.7%)	12 (10.0%)
Control 2	64 (6.7%)	62 (6.5%)	24.2	25.3	6 (9.4%)	9 (13.9%)
Control 3	70 (7.3%)	68 (7.1%)	19.1	17.0	3 (4.3%)	3 (4.3%)
Control 4	46 (4.8%)	40 (4.2%)	7.7	7.2	0	0
Control 5	22 (2.3%)	20 (2.1%)	12.1	14.8	1 (4.6%)	1 (4.6%)
Control 6	8 (0.8%)	8 (0.8%)	12.7	9.4	1 (12.5%)	0
Control 7	57 (5.9%)	55 (5.7%)	14.1	11.4	3 (5.3%)	2 (3.5%)

© 2026 Busby-Whitehead J et al. *JAMA Network Open*. This is an open access article distributed under the terms of the CC-BY-NC-ND License, which does not permit alteration or commercial use, including those for text and data mining, AI training, and similar technologies.

eTable5. Intervention Effectiveness for Secondary Outcomes

**Opioids (n=961)**	**Secondary Outcome: Discontinuation (odds ratio, 95% CI)**	**p-value**	**Secondary Outcome: Falls (odds ratio, 95% CI)**	**p-value**
Unadjusted	1.04 (0.72, 1.49)	0.82	1.04 (0.95, 1.83)	0.10
Adjusted for baseline MMEs only	1.15 (0.83, 1.59)	0.40	1.15 (0.98, 1.91)	0.06
Primary Analytic Model: Adjusted for baseline MMEs + Demographics*	1.20 (0.85, 1.71)	0.30	1.36 (0.94, 1.96)	0.10
**Benzodiazepines (n=1107)**	**Secondary Outcome: Discontinuation (odds ratio, 95% CI)**	**p-value**	**Secondary Outcome: Falls (odds ratio, 95% CI)**	**p-value**
Unadjusted	1.24 (0.85, 1.81)	0.27	1.15 (0.69, 1.92)	0.58
Adjusted for baseline DMEs only	1.40 (0.92, 2.13)	0.11	1.14 (0.69, 1.89)	0.61
Primary Analytic Model: Adjusted for baseline DMEs + Demographics*	1.41 (0.94, 2.03)	0.07	1.19 (0.67, 2.10)	0.55

© 2026 Busby-Whitehead J et al. *JAMA Network Open*. This is an open access article distributed under the terms of the CC-BY-NC-ND License, which does not permit alteration or commercial use, including those for text and data mining, AI training, and similar technologies.

eTable6. Average Daily Diazepam Milligram Equivalents by Clinic (pre- and post-intervention)

Clinic Name	Receiving Any BZDs Pre-intervention n=1107	Receiving Any BZDs Post-intervention n=1029	Average daily DMEs Pre-Intervention	Average daily DMEs Post-Intervention	DMEs >10 Pre-Intervention	DMEs >10 Post-Intervention
Intervention 1	54 (4.9%)	48 (4.3%)	10.3	10.5	16 (29.6%)	20 (37.0%)
Intervention 2	155 (14.0%)	142 (12.8%)	9.6	7.6	47 (30.3%)	37 (23.9%)
Intervention 3	87 (7.9%)	78 (7.1%)	7.3	7.8	15 (17.2%)	16 (18.4%)
Intervention 4	31 (2.8%)	31 (2.8%)	6.8	4.8	4 (12.9%)	2 (6.5%)
Intervention 5	54 (4.9%)	52 (4.7%)	8.2	8.7	16 (29.6%)	17 (31.5%)
Intervention 6	46 (4.2%)	44 (3.9%)	8.3	5.9	12 (26.1%)	10 (21.7%)
Intervention 7	50 (4.5%)	45 (4.1%)	5.2	4.8	5 (10.0%)	5 (10.0%)
Intervention 8	59 (5.3%)	57 (5.2%)	12.4	10.5	22 (37.3%)	20 (33.9%)
Control 1	102 (9.2%)	96 (8.7%)	7.1	7.2	15 (14.7%)	16 (15.7%)
Control 2	147 (13.3%)	139 (12.6%)	6.9	6.4	27 (18.4%)	24 (16.3%)
Control 3	84 (7.6%)	73 (6.6%)	5.5	5.0	13 (15.5%)	9 (10.7%)
Control 4	102 (9.2%)	98 (8.9%)	6.4	6.8	17 (16.7%)	17 (16.7%)
Control 5	29 (2.6%)	28 (2.5%)	3.8	5.8	1 (3.5%)	4 (13.8%)
Control 6	42 (3.8%)	39 (3.5%)	8.7	8.8	10 (23.8%)	10 (23.8%)
Control 7	65 (5.9%)	59 (5.3%)	7.0	6.1	9 (13.9%)	7 (10.8%)

© 2026 Busby-Whitehead J et al. *JAMA Network Open*. This is an open access article distributed under the terms of the CC-BY-NC-ND License, which does not permit alteration or commercial use, including those for text and data mining, AI training, and similar technologies.

eTable7. Effectiveness of Opioid Deprescribing Intervention on Average Daily Morphine Milligram Equivalents - High vs. Low baseline exposure

**Opioids (n=863)<br><50 MMEs per day**	**Effect Size<br>(intervention vs. control)**	**95% Confidence Intervals**	**p-value**
Unadjusted<br>(model-based SE)	0.12	(-0.10, 0.35)	0.27
Adjusted for baseline MMEs only<br>(model-based SE)	-0.02	(-0.13, 0.08)	0.66
Adjusted for baseline MMEs<br>+ Demographics*<br>(model-based SE)	-0.02	(-0.12, 0.08)	0.68
**Opioids (n=98)<br>>50 MMEs per day**	**Effect Size<br>(intervention vs. control)**	**95% Confidence Intervals**	**p-value**
Unadjusted<br>(model-based SE)	0.04	(-0.43, 0.52)	0.85
Adjusted for baseline MMEs only<br>(model-based SE)	-0.05	(-0.15, 0.04)	0.28
Adjusted for baseline MMEs<br>+ Demographics*<br>(model-based SE)	0.007	(-0.10, 0.12)	0.89

*Age, sex, race, ethnicity, Medicaid

© 2026 Busby-Whitehead J et al. *JAMA Network Open*. This is an open access article distributed under the terms of the CC-BY-NC-ND License, which does not permit alteration or commercial use, including those for text and data mining, AI training, and similar technologies.

eTable8. Effectiveness Benzodiazepine Deprescribing Intervention on Average Daily Diazepam Milligram Equivalents – High vs. low baseline exposure

**BZDs (n=893)<br><10 DMEs per day**	**Effect Size<br>(intervention vs. control)**	**95% Confidence Intervals**	**p-value**
Unadjusted<br>(model-based SE)	-0.18	(-0.27, -0.08)	0.0004
Adjusted for baseline DMEs only<br>(model-based SE)	-0.23	(-0.36, -0.09)	0.0009
Adjusted for baseline DMEs<br>+ Demographics*<br>(model-based SE)	-0.22	(-0.36, -0.08)	0.002
**BZDs (n=214)<br>>10 DMEs per day**	**Effect Size<br>(intervention vs. control)**	**95% Confidence Intervals**	**p-value**
Unadjusted<br>(model-based SE)	0.07	(-0.07, 0.22)	0.32
Adjusted for baseline DMEs only<br>(model-based SE)	-0.004	(-0.10, 0.09)	0.93
Adjusted for baseline DMEs<br>+ Demographics*<br>(model-based SE)	-0.01	(-0.11, 0.09)	0.83

*Age, sex, race, ethnicity, Medicaid

© 2026 Busby-Whitehead J et al. *JAMA Network Open*. This is an open access article distributed under the terms of the CC-BY-NC-ND License, which does not permit alteration or commercial use, including those for text and data mining, AI training, and similar technologies.

eTable9. Effectiveness of Opioid Deprescribing Intervention on Average Daily Morphine Milligram Equivalents – Older or younger than 80 years of age

**Opioids (n=248)**<br>**≥ 80 years old**	**Effect Size**<br>**(intervention vs. control)**	**95% Confidence Intervals**	**p-value**
Unadjusted<br>(model-based SE)	0.24	(-0.36, 0.84)	0.43
Adjusted for baseline MMEs only<br>(model-based SE)	-0.05	(-0.17, 0.06)	0.38
Adjusted for baseline MMEs<br>+ Demographics*<br>(model-based SE)	-0.04	(-0.17, 0.08)	0.48
**Opioids (n=713)**<br>**< 80 years old**	**Effect Size**<br>**(intervention vs. control)**	**95% Confidence Intervals**	**p-value**
Unadjusted<br>(model-based SE)	0.30	(-0.16, 0.77)	0.20
Adjusted for baseline MMEs only<br>(model-based SE)	0.07	(-0.12, 0.26)	0.48
Adjusted for baseline MMEs<br>+ Demographics*<br>(model-based SE)	0.04	(-0.15, 0.24)	0.67

*Age, sex, race, ethnicity, Medicaid

© 2026 Busby-Whitehead J et al. *JAMA Network Open*. This is an open access article distributed under the terms of the CC-BY-NC-ND License, which does not permit alteration or commercial use, including those for text and data mining, AI training, and similar technologies.
