# Supplementary material for: A Pharmacist Consultant Service for Deprescribing Opioids and Benzodiazepines in Older Adults: A Cluster Randomized Trial
Source: JAMA Netw Open. 2026 Feb 26;9(2):e2560581. doi: 10.1001/jamanetworkopen.2025.60581 (PMC12947021; doi:10.1001/jamanetworkopen.2025.60581)
Supplement: Supplement 3. — Data Sharing Statement [file jamanetwopen-e2560581-s003.pdf]

Data Sharing Statement

Busby-Whitehead. A Pharmacist Consultant Service for Deprescribing Opioids and Benzodiazepines in Older Adults. *JAMA Netw Open*. Published February 26, 2026. doi:10.1001/jamanetworkopen.2025.60581

Data
Additional Information: Clinicaltrials.gov Identifier: NCT04272671
Data available: No
